# Dexras1 is a homeostatic regulator of exercise-dependent proliferation and cell survival in the hippocampal neurogenic niche

**DOI:** 10.1038/s41598-018-23673-z

**Published:** 2018-03-28

**Authors:** Pascale Bouchard-Cannon, Christopher Lowden, Dennison Trinh, Hai-Ying M. Cheng

**Affiliations:** 1 Department of Biology, University of Toronto Mississauga, 3359 Mississauga Road, Mississauga, ON L5L 1C6 Canada; 2 Department of Cell and Systems Biology, University of Toronto, 25 Harbord Street, Toronto, ON M5S 3G5 Canada

## Abstract

Adult hippocampal neurogenesis is highly responsive to exercise, which promotes the proliferation of neural progenitor cells and the integration of newborn granule neurons in the dentate gyrus. Here we show that genetic ablation of the small GTPase, Dexras1, suppresses exercise-induced proliferation of neural progenitors, alters survival of mitotic and post-mitotic cells in a stage-specific manner, and increases the number of mature newborn granule neurons. Dexras1 is required for exercise-triggered recruitment of quiescent neural progenitors into the cell cycle. Pharmacological inhibition of NMDA receptors enhances SGZ cell proliferation in wild-type but not *dexras1*-deficient mice, suggesting that NMDA receptor-mediated signaling is dependent on Dexras1. At the molecular level, the absence of Dexras1 abolishes exercise-dependent activation of ERK/MAPK and CREB, and inhibits the upregulation of NMDA receptor subunit NR2A, *bdnf*, *trkB* and *vegf-a* expression in the dentate gyrus. Our study reveals Dexras1 as an important stage-specific regulator of exercise-induced neurogenesis in the adult hippocampus by enhancing pro-mitogenic signaling to neural progenitor cells and modulating cell survival.

## Introduction

The term “neurogenesis” refers to the process by which new neurons are generated from neural progenitor cells. Although most neurons in the mammalian brain are born during embryonic development, there are niches in the adult brain that retain the potential to undergo neurogenesis^1^. One such neurogenic niche is the subgranular zone (SGZ) of the hippocampal dentate gyrus (DG), which contains a pool of quiescent, pluripotent, radial glia-like progenitor cells, or type-1 cells^2–4^. Neurogenesis begins with the activation of type-1 cells and their recruitment into the cell cycle. Their progeny are highly proliferative and referred to as type-2 cells^5^. These are further subdivided into two stages, type-2a and type-2b, which are distinguished by the expression of glia-like (e.g., Sox2) or early neuronal markers (e.g, doublecortin [DCX]), respectively^6^. Type-2b cells give rise to lineage-committed neuroblasts, or type-3 cells, which undergo a final round of cell division before exiting the cell cycle and becoming immature neurons^7^. Over several weeks, these cells will mature, migrate and functionally integrate into existing DG circuits as newly generated DG granule neurons^8^. Of note, the majority of neural precursors do not survive to the stage of functional integration, dying instead by apoptosis during their progression through the neurogenic program^9,10^.

Adult hippocampal neurogenesis is a highly dynamic process: even though it occurs in the mammalian brain under basal conditions, the rate of neurogenesis is sensitive to an array of external factors, including aging, drugs, diseases, and social and environmental contexts^7,11–15^. One of the most robust neurogenic stimulants for laboratory rodents is voluntary exercise on a running wheel^16^. Under exercise conditions, neural progenitors and neural precursors in the SGZ receive extracellular signals from neighbouring cells, including trophic factors (e.g., brain-derived neurotrophic factor [BDNF], vascular endothelial growth factor [VEGF]) and neurotransmitters (e.g., glutamate, gamma-aminobutyric acid [GABA], serotonin, endocannabinoids), driving increases in proliferation and neuronal maturation^17,18^. The upregulation of pro-mitogenic signals not only induces proliferation but also suppresses apoptotic pathways, enhancing cell survival and the number of newborn neurons that ultimately integrate into DG circuits^19^. Pharmacological and genetic studies have implicated several receptor signaling cascades in exercise-mediated enhancement of adult hippocampal neurogenesis^18–20^.

Dexras1 (Dexamethasone-induced Ras-related protein 1), a small GTPase, has been shown to modulate several signaling cascades that are relevant to hippocampal neurogenesis. Dexras1 has intrinsic guanine nucleotide exchange factor (GEF) activity for the G_i_ subfamily of heterotrimeric G proteins, competing with GPCRs for their activation^21^. As a consequence, Dexras1 can inhibit GPCR-G_i_-mediated activation of downstream effectors including extracellular signal-regulated kinase (ERK)/mitogen-activated protein kinase (MAPK) and cAMP response element binding protein (CREB)^22–24^. There is also evidence to suggest that Dexras1 can promote the basal activity of G_i_ proteins and effectors independent of GPCR activation^25^. Other studies have linked Dexras1 to N-methyl-D-aspartate (NMDA) receptor-neuronal nitric oxide synthase (nNOS) signaling^26,27^. Dexras1 couples to NMDA receptor activation through nitrosylation by nNOS, and serves as an effector of NO signaling^26,28^. In this context, Dexras1 has been implicated in glutamate-/NO-mediated cell death and cellular uptake of iron^27,29,30^. Still other studies have linked Dexras1 to the receptor tyrosine kinase, insulin-like growth factor-1 receptor, coupling its activation to the ERK/MAPK pathway^31^.

Given the diverse functions of Dexras1 in signaling pathways relevant to neurogenesis, we asked whether ablation of *dexras1* would affect adult hippocampal neurogenesis under basal and exercise-induced conditions. We found that the DG of exercised *dexras1*^*−/−*^ mice displayed diminished rates of cell proliferation but greater numbers of newborn neurons relative to wild-type controls. Voluntary exercise triggered a two-fold increase in the number of type-1 cells that entered the cell cycle in wild-type mice but not *dexras1*^*−/−*^ animals. In these mutant mice, enhanced survival of early-dividing progenitor cells and immature neurons compensated for the reduction in SGZ cellular proliferation and absence of exercise-enhanced recruitment of type-1 cells into the cell cycle. Furthermore, *dexras1* ablation abolished exercise-dependent upregulation of p-ERK, p-CREB, NMDA receptor subunit 2A (NR2A), *bdnf*, *tropomyosin receptor kinase B* (*trkB)* and *vegf-a* in the DG. Together, our results identify Dexras1 as an important modulator of exercise-dependent neurogenesis in the murine hippocampus.

## Results

### *Dexras1* ablation suppresses exercise-induced SGZ progenitor cell proliferation but promotes retention of newborn neurons in the DG

**Figure 1:**
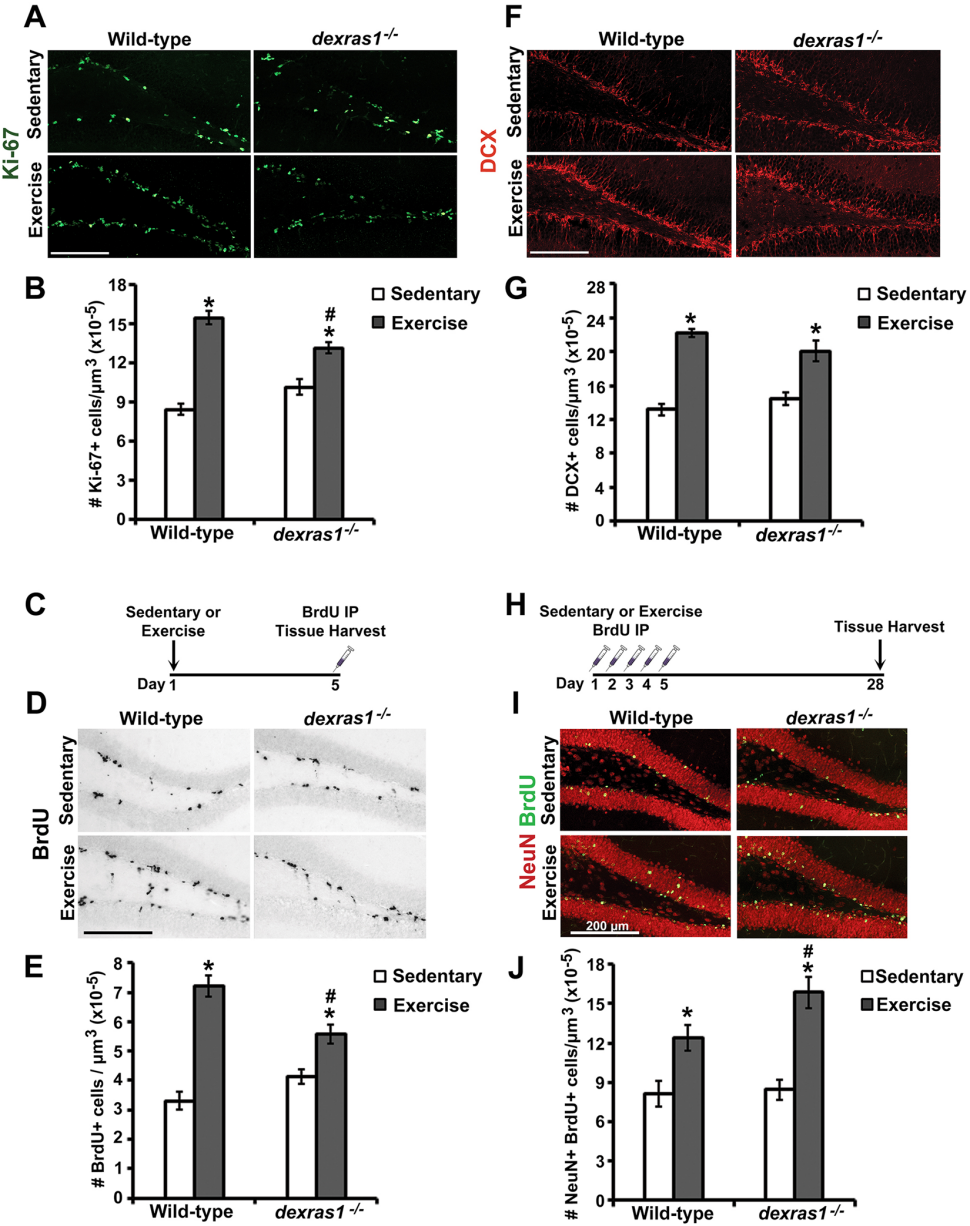
Dexras1 regulates exercise-induced proliferation of SGZ progenitor cells and survival of newborn neurons. (**A**) Representative photomicrographs of Ki-67^+^ cells (green) in the SGZ of wild-type and *dexras1*^*−/−*^ mice after 5 days of sedentary or exercise condition. Images are represented as a collapsed z-stack project (30-μm) acquired at 40× magnification. **(B)** Quantification of the number of Ki-67^+^ cells per μm^3^ of SGZ (x10^−5^). **(C)** Single BrdU-injection paradigm. Wild-type and *dexras1*^*−/−*^ mice received a single BrdU injection after 5 days of sedentary or exercise condition, and tissues were harvested 1 hr post-injection. **(D)** Representative photomicrographs of BrdU^+^ cells (black) at day 5 of sedentary or exercise condition acquired at 10× magnification. (**E**) Quantification of the number of BrdU^+^ cells per μm^3^ of SGZ (×10^−5^). **(F)** Representative photomicrographs of DCX^+^ cells (red) from wild-type and *dexras1*^*−/−*^ mice following 14 days of sedentary or exercise conditions. Images are represented as a single z-stack image (5-μm) acquired at 40× magnification. **(G)** Density of DCX^+^ cells in and adjacent to the SGZ. An area encompassing 2× the thickness of the SGZ was drawn and immunoreactive cells within this region were counted. **(H)** Label-retaining assay paradigm. Wild-type and *dexras1*^*−/−*^ mice received single daily injections of BrdU on days 1 to 5 of sedentary or exercise condition. Tissues were harvested 28 days after the first BrdU injection. **(I)** Representative photomicrographs of BrdU^+^ cells (green) and NeuN^+^ cells (red) in the DG. Images are represented as a collapsed z-stack project (30-μm) acquired at 40× magnification. **(J)** Quantification of the number of BrdU^+^NeuN^+^ cells per μm^3^ of DG (x10^−5^). Scale bar = 200 μm. All values represent mean ± standard error. *p < 0.05 vs. sedentary control. ^#^*p* < 0.05 vs. wild-type control. n = 5–7 per group.

To delineate the role of Dexras1 in adult hippocampal neurogenesis, we exposed wild-type (WT) and *dexras1*^*−/−*^ (KO) mice to short-term (5 days) exercise conditions by providing them with a running wheel in their home cage. Compared to wild-type controls under sedentary (SED, no wheel) conditions, voluntary exercise (VEx) triggered a 0.74-fold (WT VEx (1.5 × 10^−4^ ± 5.2 × 10^−6^) relative to WT SED (8.4 × 10^−5^ ± 4.4 × 10^−6^)) increase in the number of proliferating cells in the SGZ of wild-type mice, as indicated by the expression of the proliferation marker, Ki-67 (Fig. 1A,B). In contrast, exercise-mediated enhancement of SGZ cell proliferation in *dexras1*^*−/−*^ mice was attenuated (0.31-fold increase in Ki-67^+^ cells in KO Vex (1.3 × 10^−4^ ± 4.2 × 10^−6^) relative to KO SED (1.0 × 10^−4^ ± 6.0 × 10^−6^)) (Fig. 1A,B), despite their level of wheel running activity being similar to wild-type mice (WT VEx: 1.89 ± 0.07 km per day; KO VEx: 1.86 ± 0.15 km per day)^32^. Consistent with the dampened fold induction, the number of Ki-67^+^ cells was lower in exercised *dexras1*^*−/−*^ mice relative to exercised wild-type controls (Fig. 1A,B). To corroborate the Ki-67 results, we further evaluated cell proliferation using bromo-deoxyuridine (BrdU), a thymidine analog that is incorporated into DNA of proliferating cells when they are in the DNA replication phase (i.e., S-phase) of the cell cycle. After 5 days of voluntary exercise, a single intraperitoneal injection of BrdU was administered and tissues were harvested 1 hr later to assess BrdU incorporation (Fig. 1C). Exercise triggered a robust increase in the number of proliferating, S-phase cells in the SGZ of wild-type mice (1.18-fold increase in BrdU^+^ cells in WT VEx (7.2 × 10^−5^ ± 3.6 × 10^−6^) relative to WT SED (3.3 × 10^−5^ ± 3.0 × 10^−6^)) (Fig. 1D,E). The proliferation-enhancing effects of exercise were strongly diminished in *dexras1*^*−/−*^ mice (0.35-fold increase in BrdU^+^ cells in KO Vex (5.6 × 10^−5^ ± 3.3 × 10^−6^) relative to KO SED (4.1 × 10^−5^ ± 2.4 × 10^−6^)) (Fig. 1D,E). This was further reflected in significantly fewer numbers of BrdU^+^ cells in the SGZ of exercised *dexras1*^*−/−*^ mice relative to exercised wild-type controls (Fig. 1D,E). These results indicate that *dexras1* ablation dampens exercise-induced cell proliferation in the SGZ.

Next, we analyzed the total number of type-2b/-3 cells and immature neurons in the dentate gyrus after 14 days of voluntary exercise using doublecortin (DCX) as a neuronal lineage marker. Despite reduced levels of cell proliferation in the SGZ of *dexras1*^*−/−*^ mice in response to voluntary exercise (Fig. 1B,E), exercise-induced expansion of the DCX^+^ cell population appeared to be unaffected in *dexras1*^*−/−*^ mice (Fig. 1F,G). The number of DCX^+^ cells did not differ significantly between genotypes under sedentary or exercise conditions, and both genotypes exhibited a comparable increase in response to exercise (0.67-fold increase in DCX^+^ cells in WT VEx (2.2 × 10^−4^ ± 4.6 × 10^−6^) relative to WT SED (1.3 × 10^−4^ ± 6.7 × 10^−6^); 0.47-fold increase in DCX^+^ cells in KO Vex (2.0 × 10^−4^ ± 1.2 × 10^−5^) relative to KO SED (1.4 × 10^−4^ ± 7.1 × 10^−6^)) (Fig. 1F,G). Given the discrepancy between the effects of *dexras1* ablation on exercise-induced cell proliferation and expansion of neuronal precursors, we sought to determine whether progression of newborn cells to mature neurons was impacted in the knockouts. Using a label-retaining paradigm, we labeled proliferating cells with once-daily injections of BrdU for 5 consecutive days, and assessed their progression to mature neurons by quantifying the number of BrdU^+^ cells that expressed the neuronal marker NeuN within the DG 4 weeks later (Fig. H). Compared to sedentary controls, exercised mice of both genotypes had a greater number of newborn mature neurons (0.52-fold increase in BrdU^+^NeuN^+^ cells in WT VEx (1.2 × 10^−5^ ± 1.0 × 10^−6^) relative to WT SED (8.1 × 10^−6^ ± 1.0 × 10^−7^); 0.88-fold increase in BrdU^+^NeuN^+^ cells in KO Vex (1.6 × 10^−5^ ± 1.2 × 10^−6^) relative to KO SED (8.4 × 10^−6^ ± 7.7 × 10^−7^)) (Fig. 1I,J). Interestingly, the BrdU^+^NeuN^+^ population under voluntary exercise condition was significantly larger in *dexras1*^*−/−*^ mice compared to wild-type animals (Fig. 1I,J). Collectively, our data reveal that the ablation of *dexras1* promotes the production of mature DG granule neurons even as it inhibits proliferation of SGZ neural progenitors.

### *Dexras1* ablation reduces the proliferative capacity of SGZ progenitors and alters cell survival in a stage-specific manner

**Figure 2:**
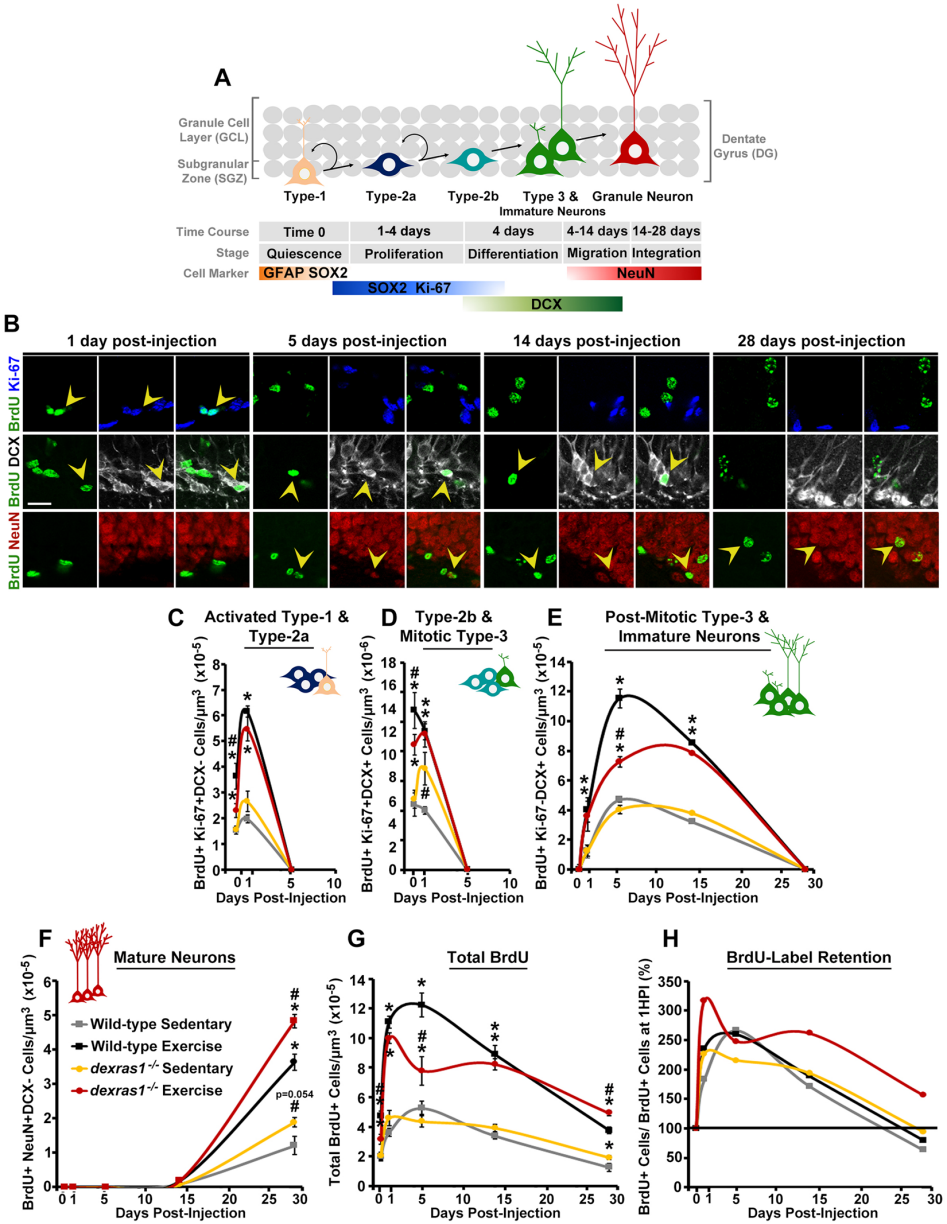
*Dexras1* ablation alters the proliferative and survival properties of progenitor cells in a stage-specific manner. **(A)** Graphical representation of hippocampal neurogenesis and respective markers used to identify the various stage-specific cell types. **(B–H)** Wild-type and *dexras1*^*−/−*^ mice were injected with a single dose of BrdU on day 5 of sedentary or exercise condition. Tissues were harvested after 1 hr (0 DPI) or after 1, 5, 14 or 28 days-post injection (DPI). **(B)** Representative photomicrographs showing the BrdU label (green) in cells positive for Ki-67 (blue), DCX (white) or NeuN (red) at 1, 5, 14 or 28 DPI. Yellow arrowheads indicate cells with co-localized expression. Images are represented as a single z-stack image (5-μm) acquired at 40× magnification. Scale bar = 20 μm. **(C–G)** Quantification of the density of BrdU-labeled **(C)** Ki-67^+^DCX^−^ activated type-1 and type-2a cells, **(D)** Ki-67^+^DCX^+^ type-2b and mitotic type-3 cells, **(E)** Ki-67^−^DCX^+^ post-mitotic type-3 cells and immature neurons, and **(F)** NeuN^+^DCX^-^ mature neurons. **(G)** Quantification of the density of all BrdU-labeled cells. X-axis indicates the number of DPI. Data are represented as mean number of cells per μm^3^ of tissue (x10^−6^ or x10^−5^) ± standard error. **(H)** Percent of BrdU-label retention. Percentage values are calculated as the number BrdU^+^ cells at each DPI divided by the number of BrdU^+^ cells at 1 hr post-injection (1 HPI). Wild-type sedentary (grey); wild-type exercise (black); *dexras1*^*−/−*^sedentary (yellow); *dexras1*^*−/−*^exercise (red). *p < 0.05 vs. sedentary control. ^#^*p* < 0.05 vs. wild-type control. n = 4–6 per group. Refer to Table S1 for cell counts.

Our aforementioned results suggest that *dexras1* deficiency may have distinct effects at different stages of adult hippocampal neurogenesis. To better understand the role of Dexras1 in adult hippocampal neurogenesis, we tracked the fate of BrdU-labeled newborn cells over a period of 28 days using stage-specific markers (Fig. 2A,B). Cell fate was assessed after 1 hr post-injection (1 HPI; as referred to as 0 DPI) or after 1, 5, 14, or 28 days post-injection (DPI). At 0 DPI, all BrdU^+^ cells are actively proliferating—most are likely still in S-phase— and therefore co-express Ki-67: these include type-2a cells and a small population of activated type-1 cells (BrdU^+^Ki67^+^DCX^−^) (Fig. 2C), as well as type-2b/mitotic type-3 cells (BrdU^+^Ki67^+^DCX^+^) (Fig. 2D). After 24 hours (1 DPI), the majority of BrdU-labeled cells are still in the proliferative stages, whereas some have become post-mitotic type-3 cells that have exited the cell cycle and ceased to express Ki-67 (Fig. 2E). The BrdU^+^Ki67^−^DCX^+^ population, which is the dominant population at 5 DPI, includes these post-mitotic type-3 cells as well as immature neurons (Fig. 2E). By 14 DPI, a very small fraction of BrdU^+^ cells have undergone further differentiation into mature neurons, losing DCX expression but retaining expression of NeuN (Fig. 2F). By 28 DPI, nearly all BrdU^+^ cells in the granule layer are mature neurons (BrdU^+^NeuN^+^DCX^−^) (Fig. 2F), except for a small subset of newly generated type-1 cells (BrdU^+^SOX2^+^GFAP^+^) in the SGZ (Fig. S1A). Only a small proportion of the initial pool of proliferating cells in the SGZ will become mature neurons, as many of the newborn cells are eliminated through apoptosis^9,33,34^. This is most evident in the decline of BrdU^+^ cell counts in wild-type animals from 5 DPI to 28 DPI (Fig. 2G).

Under sedentary conditions, neurogenesis progressed in wild-type mice in a manner similar to previous reports^34–36^. In the first 24 hr, the total pool of BrdU^+^ cells nearly doubled (Tables S1, S2, 0.83-fold increase from 1 HPI to 1 DPI), and reached a peak in cell number by 5 DPI (Fig. 2G). As the BrdU^+^ cells differentiated and migrated into the granule cell layer, their numbers declined steadily until the surviving cells were integrated into the existing neuronal network (28 DPI onwards) (Fig. 2G). Sedentary *dexras1*^*−/−*^ mice were phenotypically similar to wild-type controls, although some minor alterations were observed (Fig. S2A,C). Cell proliferation was unaffected in these animals (0 DPI in Fig. 2C,D). However, relative to wild-type controls, they showed a significant increase in the number of type-2b/mitotic type-3 cells at 1 DPI (Fig. 2D), and a near-significant increase in the number of mature neurons at 28 DPI (p = 0.054) (Fig. 2F).

Under voluntary exercise conditions, wild-type mice showed greater cell numbers throughout all stages of neurogenesis when compared to wild-type sedentary controls (Figs 2C–F, S2A,B), with the largest fold-increase in total BrdU^+^ cell numbers observed at 1 DPI and 28 DPI (~2-fold; Tables S1, S2). Despite these stark differences in total BrdU^+^ cell numbers, BrdU^+^ cells progressed through the neurogenic program with temporal profiles that were comparable between sedentary and exercised wild-type mice (Figs 2C–H, S2A,B). From 1 HPI to 1 DPI, exercised wild-type mice showed a 1.35-fold increase in BrdU^+^ cell numbers, whereas sedentary controls displayed a substantially lower fold increase of 0.83 (Fig. 2G, Tables S1, S2). This suggests that exercise not only increases the proliferative capacity of mitotic cells, but it may also enhance their survival during the stage of progenitor cell pool expansion. After reaching a peak in BrdU^+^ cell numbers at 5 DPI, exercised wild-type mice showed a steady depletion of BrdU^+^ cells until the mature neuronal stage was reached (28 DPI), at a rate comparable to that of sedentary mice (Fig. 2G,H, Tables S1, S2). Although exercised wild-type mice generated more mature granule neurons for synaptic integration into the granule cell layer, these mice showed roughly similar fold-change in BrdU^+^ cells at 28 DPI relative to 1 HPI when compared to sedentary controls (−0.20 for WT VEx; −0.37 for WT SED) (Tables S1, S2). This suggests that at the post-mitotic stage, exercise does not promote cell survival, and that the increase in BrdU^+^NeuN^+^ cells at 28 DPI is most likely due to the large expansion (i.e., increased proliferation) and survival of early mitotic cells.

By placing *dexras1*^*−/−*^ mice under voluntary exercise conditions, we noticed that these mice exhibited phenotypic differences in the proliferative and survival capacity of progenitor cells when compared to exercised wild-type controls (Figs 2C–F, S2B,D). The fold-difference in total BrdU^+^ cell numbers at 1 HPI between exercise and sedentary conditions was 1.38 for wild-type animals, but only 0.56 for *dexras1*^*−/−*^ mice (Tables S1, S2, Fig. 2G). This was further reflected in the significantly smaller pool of BrdU^+^ activated type-1/type-2a cells (Fig. 2C) and BrdU^+^ type-2b/mitotic type-3 cells (Fig. 2D) at 1 HPI in the *dexras1*^*−/−*^ mice. However, by 1 DPI, the numbers of these actively dividing cell types were comparable between wild-type and *dexras1*^*−/−*^ mice under voluntary exercise conditions (Fig. 2C,D). In line with these observations, the fold-increase in total BrdU^+^ cell numbers between 1 HPI and 1 DPI was much larger for *dexras1*^*−/−*^ mice (2.17 fold) than for wild-type animals (1.35 fold) (Tables S1, S2). These results suggest that the deletion of *dexras1* blunts the proliferative effects of exercise, but enhances the survival of early-dividing progenitor cells. Between 1 DPI and 5 DPI, when total BrdU^+^ cell numbers in exercised wild-type animals were relatively constant (0.10-fold increase), exercised *dexras1*^*−/−*^mice showed a 0.22-fold decrease in BrdU^+^ cell count (Tables S1, S2). Moreover, during this time window the post-mitotic type-3 cell/immature neuron population increased by 1.89-fold in exercised wild-type mice, but only by 1.02-fold in *dexras1*^*−/−*^ mice, culminating in a significantly smaller population of these cells at 5 DPI in *dexras1*^*−/−*^ mice compared to wild-type controls (Fig. 2E). These results suggest that, under voluntary exercise conditions, the ablation of *dexras1* may attenuate the survival of newly post-mitotic cells. Between 5 DPI and 14 DPI, when exercised wild-type mice experienced a significant loss of post-mitotic type-3 cells/immature neurons, there was no observable change in the number of these cells in *dexras1*^*−/−*^ mice (Fig. 2E). During the maturation phase, Dexras1 may therefore be a major contributor to cell selection prior to neuronal network integration. From 14 DPI to 28 DPI, exercised *dexras1*^*−/−*^ mice, similar to all other experimental groups, showed a downward slope in total BrdU^+^ cell numbers (Fig. 2G). Nonetheless, exercised *dexras1*^*−/−*^mice had significantly more mature neurons (BrdU^+^NeuN^+^) in the DG at 28 DPI than any other experimental group (Fig. 2F). This phenotype is particularly notable given that the initial population of proliferating cells that were labeled by BrdU was much smaller in exercised *dexras1*^*−/−*^ mice compared to wild-type controls. The fold-change in total BrdU^+^ cell numbers from 1 HPI to 28 DPI was 0.56 for exercised *dexras1*^*−/−*^ mice and −0.20 for wild-type controls (Fig. 2H, Tables S1, S2). Notably, altered fate of newborn cells could not explain the increase in BrdU^+^ neurons in exercised *dexras1*^*−/−*^ mice relative to all other experimental groups, since we found no colocalization of BrdU with the astrocyte marker, S100β, within the DG at 28 DPI in any group, suggesting that the majority of labeled precursor cells undertook a neuronal cell fate (Fig. S1B).

Collectively, our results suggest that *dexras1* deficiency has multiple, stage-specific effects on exercise-induced adult hippocampal neurogenesis, but that it ultimately enhances the number of mature neurons that are produced. *Dexras1* deficiency reduces cell proliferation in the SGZ, and potentially regulates cell survival in a bidirectional and stage-dependent manner.

### Dexras1 is required for exercise-induced recruitment of quiescent neural progenitors into the cell cycle

Several hypotheses have been proposed to explain the proliferation-enhancing effects of exercise on hippocampal neural progenitors^37^. These include: (1) recruitment of quiescent neural progenitors into the cell cycle; (2) acceleration of the cell cycle; (3) increased number of cell divisions; and (4) attenuation of cell death^37^. The BrdU label retention experiment described above (Fig. 2G, Tables S1, S2) provides evidence to suggest that exercise enhances the survival of proliferating cells in the DG, but that the combination of exercise and *dexras1* ablation modulates mitotic and post-mitotic cell survival either positively or negatively, depending on the stage of neurogenesis. The rapid clearance of dying cells by microglia and immature neuroblasts in the DG renders any attempt to identify and quantify dying cells with conventional techniques, such as cleaved caspase-3 or TUNEL labeling, extremely challenging^38–40^.

**Figure 3:**
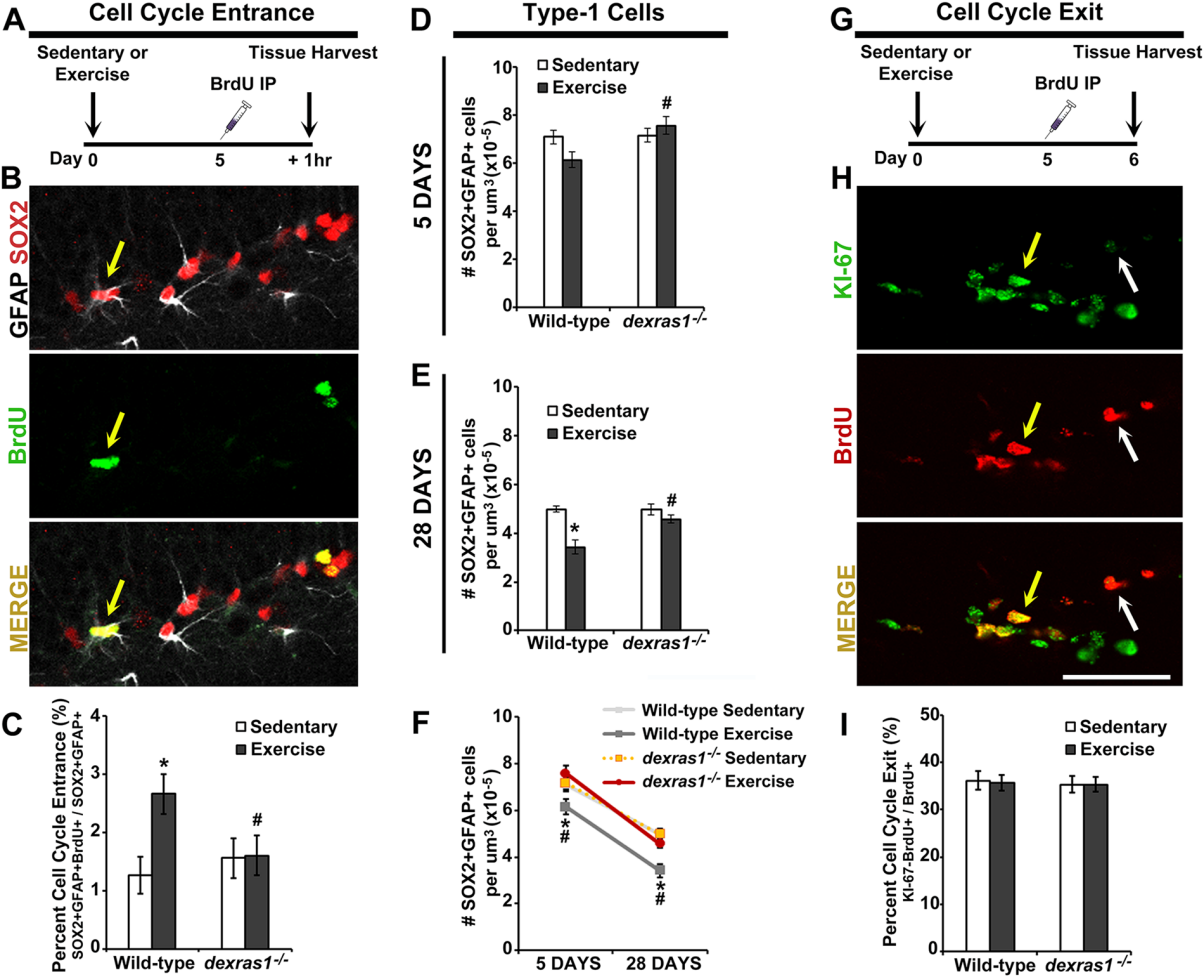
Dexras1 is required for exercise-mediated recruitment of type-1 cells into the cell cycle. (**A**) BrdU-injection paradigm for cell cycle entrance. Wild-type and *dexras1*^*−/−*^ mice were placed under sedentary or exercise condition, injected once with BrdU on day 5, and killed for tissue after 1 hr post-injection. **(B)** Representative photomicrographs of BrdU-labeled (green) cells that co-express GFAP (white) and SOX2 (red). **(C)** Percent of type-1 cells that recently entered the cell cycle. Values are calculated by dividing the number of BrdU^+^GFAP^+^SOX2^+^ cells by the total number of GFAP^+^SOX2^+^ cells in the SGZ. **(D–F)** Quantification of the number of SOX2^+^GFAP^+^ cells per μm^3^ of SGZ (x10^−5^) after **(D)** 5 days or **(E)** 28 days of sedentary or exercise condition. **(F)** shows the combined data of both time points. **(G)** BrdU-injection paradigm for cell cycle exit. Wild-type and *dexras1*^*−/−*^ mice were injected once with BrdU on day 5 of sedentary or exercise conditions, and killed for tissue after 24 hr post-injection. **(H)** Representative photomicrographs of BrdU-labeled (red) cells that co-express Ki-67 (green). **(I)** Percent of proliferating cells that have exited the cell cycle after 24 hr. Values are calculated by dividing the number of BrdU^+^Ki-67^−^ cells by the total number of BrdU^+^ cells in the SGZ. All values represent mean ± standard error. *p < 0.05 vs. sedentary control. ^#^*p* < 0.05 vs. wild-type control. n = 5–6 per group. Yellow arrows indicate cells with co-localized expression; white arrow shows a BrdU^+^Ki-67^−^ cell. Images are represented as a single z-stack image (5-μm) acquired at 40× magnification. Scale bar = 50 μm.

To assess whether the proposed cell cycle-related mechanisms also contribute to the neurogenesis phenotype of *dexras1*^*−/−*^ mice, we used different thymidine analog injection paradigms to evaluate cell cycle entrance, cell cycle kinetics, and cell cycle exit. First, we determined whether exercise or *dexras1* deficiency affected the recruitment of type-1 cells into the cell cycle by injecting mice with BrdU and quantifying the number of BrdU^+^Sox2^+^GFAP^+^ cells 1 hr post-injection (Fig. 3A,B). Relative to sedentary controls, wild-type mice under exercise conditions exhibited a 2-fold increase in the percentage of type-1 cells that were actively proliferating (Fig. 3C). In contrast, exercise had no effect on the percentage of proliferating type-1 cells in *dexras1*^*−/−*^ mice (Fig. 3C). These data suggest that Dexras1 is required for exercise-induced upregulation of cell cycle entrance of type-1 cells. To further substantiate this interpretation, we examined the effects of exercise or *dexras1* ablation on the size of the type-1 pool. Enhanced recruitment of type-1 cells should result in accelerated depletion of the type-1 pool in exercised wild-type, but not *dexras1*^*−/−*^, mice. Regardless of genotype or level of physical activity, the number of SOX2^+^GFAP^+^ cells was significantly reduced in 2-month-old mice (Fig. 3E-F) relative to 1-month-old mice (Fig. 3D,F), indicating a depletion of the type-1 pool as animals age. Voluntary exercise for 28 days accelerated the depletion of the type-1 pool in wild-type but not *dexras1*^*−/−*^ mice (Fig. 3E,F). Importantly, after 28 days of exercise, the type-1 pool was significantly larger in *dexras1*^*−/−*^ mice relative to wild-type controls (Fig. 3F). To determine whether exercise or *dexras1* deficiency affects the rate of cell cycle exit, we injected mice with BrdU and quantified the proportion of BrdU^+^ cells that ceased express Ki-67 24 hr post-injection (Fig. 3G,H). Neither exercise nor *dexras1* ablation altered the probability of cell cycle exit (Fig. 3I). The cell cycle exit rate can be used to estimate how many rounds of cell division neural progenitors will undergo prior to withdrawal from the cell cycle^41–43^. Hence, our data suggest that the average number of cell divisions for the total pool of dividing cells is not altered by either exercise or *dexras1* ablation (Fig. 3I).

**Figure 4:**
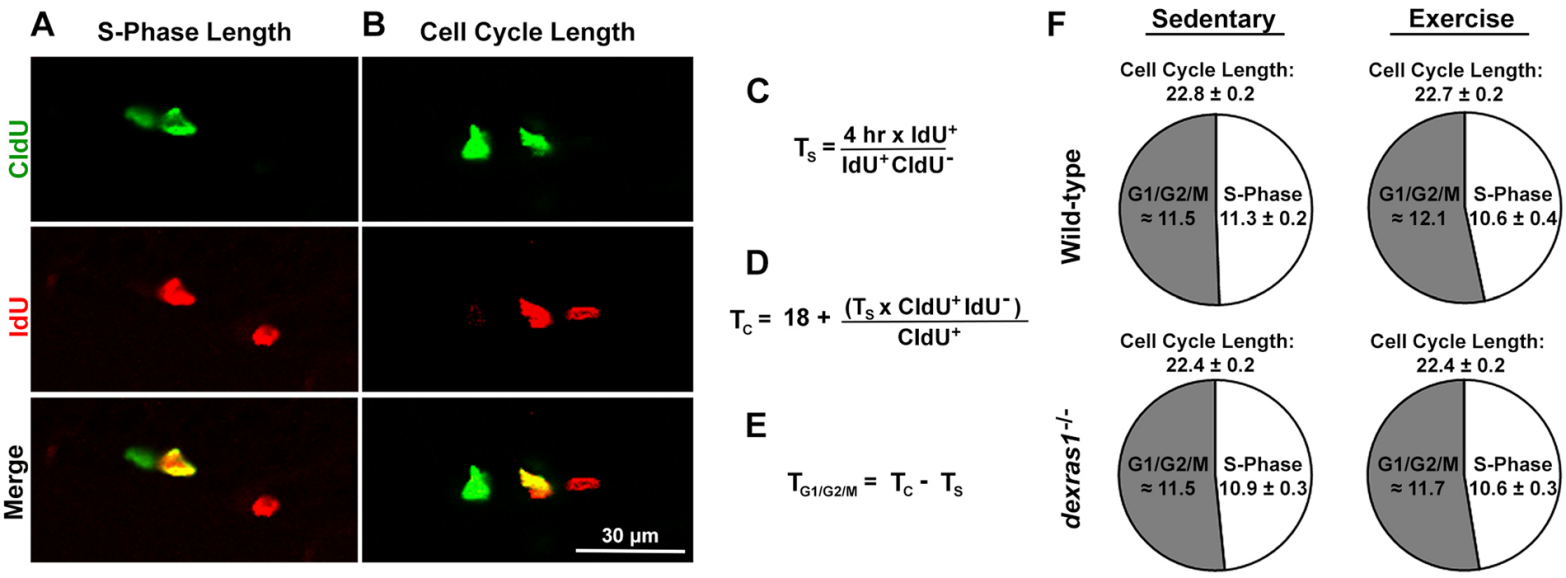
Neither *dexras1* ablation nor voluntary exercise alters the average S-phase length and cell cycle length of proliferating neural progenitor cells. Wild-type and *dexras1*^*−/−*^ mice received a single IdU injection on day 5 of sedentary or exercise condition. For S-phase length calculations, mice received a CldU injection 4 hr post-IdU injection. For cell cycle length calculations, mice received a CldU injection 18 hr post-IdU injection. Hippocampal sections were harvested 45 min post-CldU injection. (**A**,**B**) Representative photomicrographs of single- and double-labeled CldU^+^ (green) and IdU^+^ (red) cells used for the quantification of **(A)** S-phase length and **(B)** cell cycle length. Images are represented as a single z-stack image (5-μm) acquired at 40× magnification. Scale bar = 30 μm. **(C–E)** Mathematical formulas used for the calculation of **(C)** S-phase length (T_s_), **(D)** cell cycle length (T_c_), and **(E)** the estimated G1/G2/M-phase combined length. **(F)** Pie chart representation of the average cell cycle length, S-phase length and estimated G1/G2/M combined length. Values represent mean ± standard error or estimated value. n = 6–9 per group.

Next, we investigated whether exercise or *dexras1* ablation affected cell cycle kinetics in the SGZ using the mutually distinguishable thymidine analogs, iodo-deoxyuridine (IdU) and chloro-deoxyuridine (CldU), and different inter-injection intervals to measure S-phase (T_s_) and total cell cycle lengths (T_c_) of the entire pool of dividing cells (Fig. 4A–E). We found that neither exercise nor *dexras1* ablation affected mean S-phase or total cell cycle length of this mixed population (Fig. 4F).

Finally, using the injection paradigm for total cell cycle length measurement, we calculated the percentage of dividing cells that entered a second cell cycle within 18 hours, and used that as an estimate of the relative rates of cell cycle re-entrance of (predominantly) type-2 cells (Fig. S3A). We found that 19% ± 1% of dividing cells from sedentary wild-type mice re-entered the cell cycle. Exercise increased the frequency of cell cycle re-entrance to 33% ± 0.7% in wild-type mice (Fig. S3B). Compared to wild-type controls, *dexras1*^*−/−*^ mice exhibited similar rates of cell cycle re-entrance under exercise conditions (32 ± 1%) but significantly higher rates (27 ± 2%) under sedentary conditions (Fig. S3B). The cell cycle re-entrance rate that is attributed to the effects of exercise is therefore diminished in *dexras1*^*−/−*^ mice compared to wild-type animals (5% vs. 14%) (Fig. S3B). These results suggest a potential role of Dexras1 in cell cycle re-entrance of type-2 cells under sedentary conditions. This effect of exercise on type-2 cells may explain why overall cell proliferation is increased in exercised *dexras1*^*−/−*^ mice relative to sedentary controls, despite the observed abrogation in exercise-induced type-1 cell activation.

Overall, our results indicate that voluntary exercise elevates SGZ cell proliferation, in part through increased recruitment of type-1 cells into the cell cycle, a process that is dependent on Dexras1 and comes at the cost of accelerated type-1 cell depletion in the SGZ. On the other hand, cell cycle exit rate and cell cycle length of SGZ neural progenitors do not appear to be affected by the ablation of *dexras1*.

### *Dexras1* ablation suppresses exercise-dependent activation of pro-mitogenic pathways in the DG

**Figure 5:**
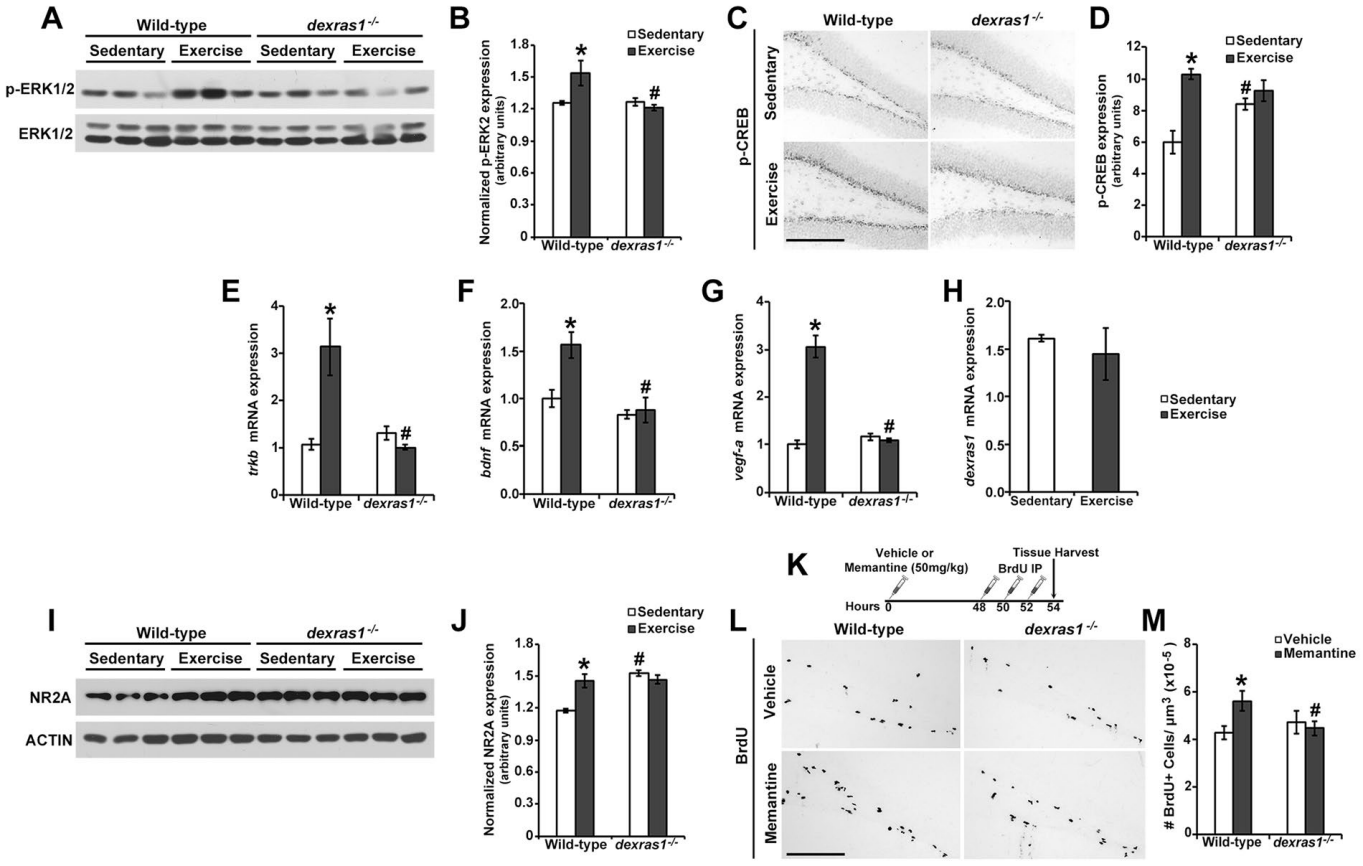
Exercise-induced activation of pro-mitogenic signaling cascades is dependent on Dexras1 expression. **(A)** Representative western blots showing the expression of phospho-ERK1/2 (p-ERK1/2) and total ERK1/2, in the DG of wild-type and *dexras1*^*−/−*^mice after 5 days of sedentary or exercise condition. p-ERK1/2 western blots were optimized for quantification of p42 (ERK2). p44 (ERK1) could be visualized with longer development times (not shown). **(B)** Quantification of the relative protein abundance of p-ERK2, normalized to total ERK1/2. n = 3 per genotype and condition. **(C)** Representative photomicrographs of phospho-CREB (p-CREB) immunoreactivity in the SGZ of wild-type and *dexras1*^*−/−*^mice after 5 days of sedentary or exercise condition. Scale bar = 200 μm. **(D)** Quantification of p-CREB immunoreactive intensity in the SGZ. n = 4–6 per group. **(E–H)** qRT-PCR analysis of the relative mRNA abundance of **(E)** *trkB*, **(F)** *bdnf*, **(G)** *vegf-a* and **(H)** *dexras1* in the DG of wild-type and *dexras1*^*−/−*^mice after 5 days of sedentary and exercise condition. Values were normalized to *gapdh* expression. n = 3–4 per genotype and condition. **(I)** Representative western blot showing the expression of NMDA Receptor 2A (NR2A) and ACTIN in the DG of wild-type and *dexras1*^*−/−*^ mice after 5 days of sedentary or exercise condition. **(J)** Quantification of the relative protein abundance of NR2A normalized to actin. n = 3 per genotype and condition. **(K)** Memantine and BrdU-injection paradigm. Sedentary wild-type and *dexras1*^*−/−*^mice were injected once with memantine or vehicle prior to receiving 3 injections of BrdU spaced 2 hr apart starting 48 hr after memantine administration. Tissues were harvested 2 hr after the last BrdU injection. **(L)** Representative photomicrographs of BrdU^+^ (black) cells in the SGZ of memantine- or vehicle-treated mice. Scale bar = 200 μm. **(M)** Quantification of the number of BrdU^+^ cells per μm^3^ of SGZ (x10^−5^) in memantine- or vehicle-treated mice. n = 4–5 per group. All values represent mean ± standard error. *p < 0.05 vs. sedentary control. ^#^*p* < 0.05 vs. wild-type control.

Voluntary exercise is known to stimulate pro-mitogenic pathways and gene transcription in the DG of the hippocampus^44^. To determine whether induction of mitogenic signaling is suppressed in the DG of exercised *dexras1*^*−/−*^ mice, thereby providing a possible mechanism for the proliferation phenotype, we assessed the expression of phospho-ERK1/2 (Fig. 5A,B) and one of its downstream effectors, CREB (Fig. 5C,D), in the DG after 5 days of sedentary or exercise conditions. Voluntary exercise triggered a significant increase in p-ERK1/2 and p-CREB^Ser133^ in the DG of wild-type mice, whereas their expression was not induced in exercised *dexras1*^*−/−*^ mice relative to sedentary controls (Fig. 5A–D). In the case of p-CREB, sedentary *dexras1*^*−/−*^ mice displayed higher basal levels of p-CREB in the DG compared to sedentary wild-type controls, and exercise did not further elevate its expression (Fig. 5C,D). p-ERK1/2 levels were comparable between sedentary wild-type and *dexras1*^*−/−*^ mice (Fig. 5A,B), suggesting that other kinases (or phosphatases) may be responsible for enhanced p-CREB expression in sedentary *dexras1*^*−/−*^ mice.

Next, we asked whether exercise affected the expression of pro-mitogenic target genes of CREB. In wild-type mice, exercise increased the expression of *bdnf*, *vegf-a*, and the BDNF receptor *trkB* at the mRNA level compared to sedentary controls (Fig. 5E–G). Their expression was comparable between sedentary wild-type and *dexras1*^*−/−*^ mice, but none were induced by voluntary exercise in *dexras1*^*−/−*^ mice (Fig. 5E–G). These results suggest that Dexras1 is required for exercise-induced pro-mitogenic gene expression in the DG. We also evaluated *dexras1* gene expression in the DG of wild-type mice, and found that voluntary exercise had no effect on levels of dexras1 transcripts (Fig. 5H).

Finally, we examined the potential effects of *dexras1* ablation on NMDA receptor-mediated glutamatergic signaling in the DG under sedentary and exercise conditions. Several studies have uncovered functional interactions between Dexras1 and NMDA receptor signaling^45–48^. Moreover, glutamatergic signaling can, either directly or indirectly (through the regulation of paracrine signals), affect cell proliferation and survival in the hippocampus^18^. After 5 days of voluntary exercise, wild-type mice exhibited a significant upregulation in the abundance of the NMDA receptor subunit NR2A (Fig. 5I,J). In contrast, exercise did not induce the expression of NR2A in *dexras1*^*−/−*^ mice, owing to the already elevated basal expression in sedentary mice (Fig. 5I,J).

To determine whether *dexras1* ablation had a functional impact on NMDA receptor-mediated signaling in the DG, we examined the effects of memantine, an NMDA receptor antagonist, on cell proliferation in the DG of wild-type and *dexras1*^*−/−*^ mice. Pre-treatment with memantine 72 hr prior to BrdU administration was previously shown to promote SGZ cell proliferation under basal conditions^49^. In our experiment, wild-type and *dexras1*^*−/−*^ mice were given a single injection of memantine or vehicle, placed in sedentary conditions for 48 hr, and subsequently given 3 injections of BrdU, spaced 2 hr apart, prior to tissue harvest 2 hr after the final BrdU injection (Fig. 5K). this injection paradigm was optimized under our laboratory conditions to elicit an effect on cell proliferation in the SGZ of wild-type mice. As previously shown by Maekawa *et al*., memantine significantly increased SGZ cell proliferation in wild-type mice by 0.25-fold relative to vehicle control (Fig. 5L,M)^49^. The proliferation-enhancing effects of memantine were completely absent in *dexras1*^*−/−*^ mice (Fig. 5L,M). Furthermore, the number of BrdU^+^ cells in memantine-treated *dexras1*^*−/−*^ mice was lower than that in memantine-treated wild-type mice (p = 0.053) (Fig. 5L,M). The ineffectiveness of memantine to promote SGZ cell proliferation in *dexras1*^*−/−*^ mice suggests that at least some aspects of NMDA receptor-mediated signaling are suppressed in *dexras1*^*−/−*^ mice, even under basal (sedentary) conditions. Collectively, our data suggest that altered mitogenic and NMDA receptor-mediated signaling may be potential mechanisms through which *dexras1* ablation affects adult hippocampal neurogenesis.

## Discussion

Numerous studies have shown that physical activity promotes adult hippocampal neurogenesis, but the underlying mechanisms are not clearly understood^37^. Here we provide evidence that Dexras1 is a regulator of exercise-induced neurogenesis in the adult murine hippocampus. Relative to wild-type controls, *dexras1*^*−/−*^ mice exhibited an attenuation in exercise-induced progenitor cell proliferation in the SGZ, but a paradoxical increase in the number of newborn mature neurons that ultimately integrated into the DG (Figs 1A–C, H–J; Fig. 2C,D,F). Our data showed that voluntary exercise enhanced the recruitment of type-1 cells into the cell cycle only in wild-type mice; the absence of this effect in *dexras1*^*−/−*^ mice may partly underlie the dampened rate of proliferation (Fig. 3A–C). By tracking the fate and number of BrdU label-retaining cells over the course of 4 weeks, we found evidence to suggest that the survival of proliferating neural progenitors and their post-mitotic progeny is altered in *dexras1*^*−/−*^ mice (Fig. 2C–H). Our data are consistent with the interpretation that *dexras1* ablation enhances the survival of early-dividing progenitor cells and maturing neurons, but inhibits the survival of newly post-mitotic cells. At the molecular level, *dexras1* ablation blocked the induction of pro-mitogenic signaling (i.e., p-ERK, p-CREB) and gene expression (i.e., *trkb*, *bdnf*, *vegf-a*) that was normally observed in the DG of wild-type mice under voluntary exercise conditions (Fig. 5A–G). NMDA receptor-mediated glutamatergic signaling in the DG also appeared to be impacted by *dexras1* ablation (Fig. 5I–M). It remains to be determined whether these changes at the molecular level are causal to the proliferation and cell survival phenotypes exhibited by *dexras1*^*−/−*^ mice, or whether they are merely correlative.

Previous label-retaining studies using sedentary mice showed that the number of newborn cells in the SGZ peaked in the first 15–48 h following BrdU injection, and gradually declined as the cells progressed through the different stages of neurogenesis^34–36^. The results from our time course study are consistent with these findings, and in addition revealed that neurogenesis progresses in a similar temporal fashion in the SGZ of wild-type mice regardless of the animal’s level of physical activity (Fig. 2G). However, under exercise conditions, wild-type mice maintained a 1.3- to 2-fold increase in BrdU^+^ cells across all stages of neurogenesis compared to their sedentary counterparts. In terms of the type-1 cells, exercise effectively doubled the number of these cells that are recruited into the cell cycle (Fig. 3A–C). Although some previous studies have suggested that type-1 cells are unresponsive to physical exercise, our results mirror those from a more recent study which observed that running triggered an increase in cell cycle entrance of quiescent radial glia-like progenitor cells^12,50,51^. The enhanced recruitment of type-1 cells would contribute to the expansion of the type-2a cell pool in exercised mice relative to sedentary controls, but it is unlikely to be the only mechanism, since we estimate that the absolute numbers of newly activated (BrdU^+^) type-1 cells represent ~5% of the total population of S-phase cells (Fig. 2C). A second mechanism may be the enhanced survival of early-dividing neural progenitor cells under exercise conditions. In the first 24 hr post-injection, the numbers of total BrdU^+^ cells and BrdU^+^ type-1/type-2a cells increased by ~1.35-fold in exercised wild-type mice but only by ~0.83-fold in sedentary controls (Table S1, S2). Several studies of basal neurogenesis have supported the notion that cell death can occur during the progenitor stages, although other studies have disputed this, suggesting that cell death, under basal conditions, is restricted to the post-mitotic population^9,33–35,52^. We found scant evidence to suggest that exercise affects the survival of post-mitotic cells in the DG, since the rate of BrdU^+^ cell loss at the later stages of neurogenesis (5 DPI to 28 DPI) was similar between sedentary and exercise conditions (Fig. 2G,H). Other potential mechanisms such as delay of cell cycle exit (to promote an additional round of cell division) and changes in cell cycle kinetics (to control the switch from proliferation to differentiation) were not apparent when we compared wild-type mice under sedentary and exercise conditions (Fig. 3G–I). In line with our results (Fig. 3A–F), Fischer *et al*. also found that running had no effect on S-phase or cell cycle length^53^. Certainly, a more detailed exploration of cell type-specific cell cycle kinetics may be needed in order to shed light on the dynamics of different subpopulations in the hippocampal niche. Our present data lead us to hypothesize that exercise elevates granule neuron production by potentially three mechanisms: (1) promoting type-1 cell activation, (2) increasing the rate of cell cycle re-entrance of type-2 cells, and (3) promoting the survival of mitotic cells in the early stage of neurogenesis. The effects of exercise are largely directed towards the expansion of the mitotic pool: the increase in the number of newly generated neurons in the granule layer is merely a consequence of this, since exercise does not appear to affect the survival of post-mitotic cells.

Importantly, our study revealed that, despite being dispensable for basal neurogenesis, the expression of Dexras1 is critical for modulating the effects of exercise on progenitor cell proliferation and mitotic cell survival. The proliferation-enhancing effects of exercise were attenuated in *dexras1*^*−/−*^ mice relative to wild-type controls, as indicated by the reduced number of BrdU^+^ S-phase cells at 1 HPI (Figs 1C–E; 2C–B). In these mutant animals, exercise had no effect on the recruitment of type-1 cells into the cell cycle, suggesting that Dexras1 is required for exercise-induced activation of quiescent type-1 neural progenitors (Fig. 3A–C). However, even though exercise-dependent type-1 cell recruitment was blocked, *dexras1*^*−/−*^ mice still exhibited a moderate increase in proliferation under exercise conditions relative to their sedentary counterparts at 1 HPI (Fig. 1C–E; Fig. 2C,D). This is likely due to the enhanced survival of early-dividing (type-2a) cells in exercised *dexras1*^*−/−*^ mice, which after 5 days of exercise (0 DPI) would lead to an enlargement of the proliferative cell pool. The combination of exercise and *dexras1* ablation was more effective than exercise alone at promoting the survival of early-dividing progenitor cells, as shown by the fold-increase in the type-2a cell population in the first 24 hr post-BrdU injection (Fig. 2C). It is also worth noting that *dexras1* ablation does not alter cell cycle kinetics or the rate of cell cycle exit (Figs 3G–I; 4A–F). Thus, a reduction in the T_s_/T_c_ ratio (leading to cells spending proportionately less time in S-phase) and/or acceleration of cell cycle exit (leading to fewer cell divisions) cannot be used to explain the proliferation phenotype of these animals.

In addition to these effects on proliferating cells, *dexras1* ablation also appeared to impact neural precursors in the later stages of neurogenesis. From 1 DPI to 5 DPI, as newborn cells progressed to the stage of post-mitotic type-3 cells and immature neurons, the large expansion of this population that we observed in exercised wild-type mice was severely blunted in *dexras1*^*−/−*^ animals (Fig. 2E). This coincided with a dip in total BrdU^+^ cell number at 5 DPI in the mutant mice (Fig. 2G). We believe that the reduced expansion of the post-mitotic pool in exercised *dexras1*^*−/−*^ mice is the result of 1) attenuated cell proliferation in the earlier stages combined with 2) reduced survival of post-mitotic type-3 neuroblasts.

During the differentiation, migration and integration stages (5 DPI to 28 DPI), the numbers of BrdU^+^ cells continued to be impacted in *dexras1*^*−/−*^ mice. From 5 DPI to 14 DPI, when the population of BrdU^+^ post-mitotic type-3 cells/ immature neurons dwindled in wild-type mice, the numbers of these cells remained constant in *dexras1*^*−/−*^ mice, suggesting that *dexras1* ablation may enhance their survival as they undergo differentiation and migration (Fig. 2E). This phenotype may be independent of exercise, since sedentary *dexras1*^*−/−*^ mice also showed a similar, if somewhat diminished, effect. Furthermore, the increase in survival of post-mitotic type-3 cells/immature neurons from 5 to 14 DPI may balance the heightened elimination of post-mitotic type-3 cells from 1 to 5 DPI, resulting in an unaltered total DCX^+^ cell pool (Fig. 1E-G). From 14 DPI onwards, as cells transitioned to the final phase of synaptic integration, exercised *dexras1*^*−/−*^ mice displayed a more gradual reduction in total BrdU^+^ cell numbers, resulting in more newborn neurons in the DG by 28 DPI (Fig. 2F,G). This increase in cell survival at the final stage of neurogenesis may be due to effects of *dexras1* ablation directly on apoptotic/ cell survival pathways, or indirectly through enhanced synaptic integration of newborn neurons. Due to their extremely low numbers and rapid clearance, apoptotic cells are difficult to quantify in the DG: thus, discrimination between these two possibilities requires an alternate approach beyond the conventional methods of using *in situ* apoptotic markers. Also, it should be noted that *dexras1* ablation affects hippocampal-dependent memory and learning under basal conditions^48^. It would be interesting to know whether the alterations in exercise-dependent hippocampal neurogenesis in *dexras1*^*−/−*^ mice impact the cognitive-enhancing effects of exercise.

**Figure 6:**
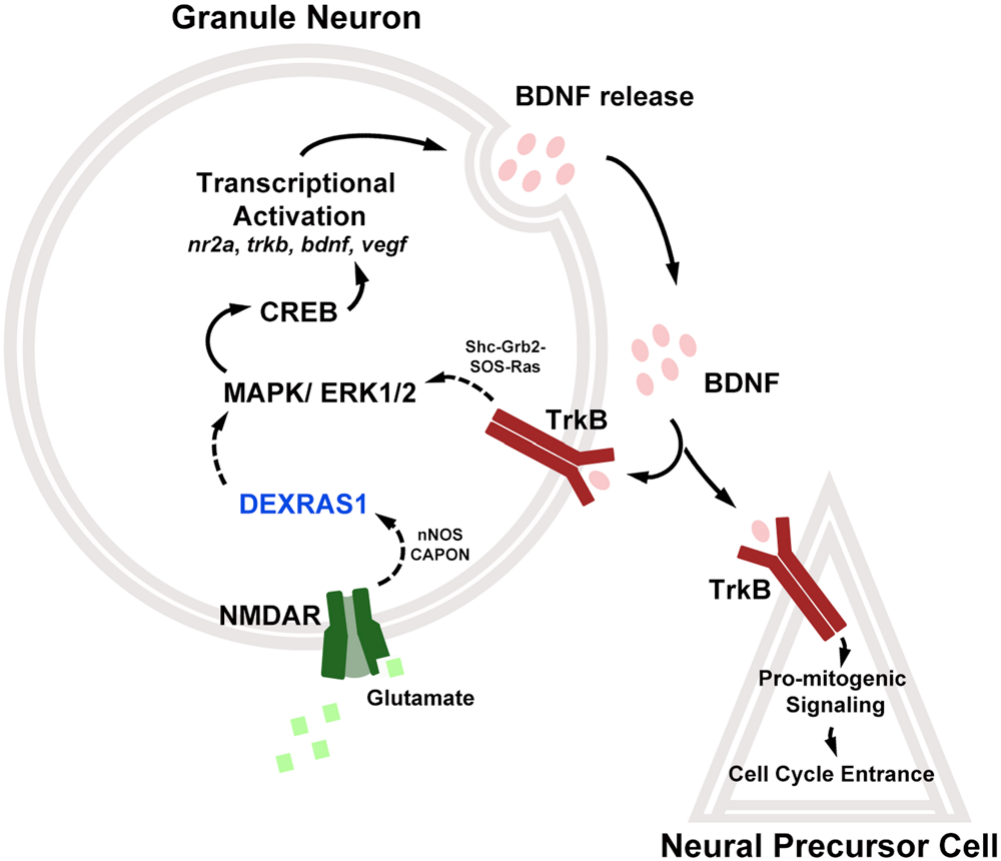
Hypothetical model for Dexras1-mediated recruitment of quiescent neural progenitor cells into the cell cycle. Hypothetical model depicting the role of Dexras1 in the regulation of exercise-induced neurogenesis. The model asserts that Dexras1 couples NMDA receptor activation to MAPK/ERK signaling in granule neurons, leading to CREB-mediated transcriptional activation of pro-mitogenic genes including *bdnf*. BDNF is released and acts on type-1 cells to promote cell cycle entrance. It can also act on granule neurons to further bolster the activity of the NMDAR-MAPK/ERK-CREB cascade.

The molecular mechanisms that are causal to the neurogenic phenotype of *dexras1*^*−/−*^ mice remain unclear and warrant further investigation. Dexras1 may be functioning in multiple cell types of the hippocampal neurogenic cascade, or even in other brain regions that send inputs to the hippocampus, to produce the observed effects on proliferation and survival of progenitor and post-mitotic cells^26,54^. In addition, Dexras1 may be coordinating the activities of multiple signal transduction pathways, with some or all of these contributing to the neurogenic phenotype. Although we have developed one hypothetical model to explain how Dexras1 may be acting within the DG to regulate cell proliferation and survival (Fig. 6; discussion below), there may be other mechanisms involved.

The pro-mitogenic effects of Dexras1 on SGZ cell proliferation may be linked to its involvement in NMDA receptor (NMDAR) and MAPK/ERK signaling^45,47^. The GEF activity of Dexras1 is induced by S-nitrosylation following activation of NMDA receptors^26,28^. Dexras1 serves as a mediator of some aspects of NMDAR signaling, including resetting of the brain’s central circadian pacemaker and iron uptake in cortical neurons^45,46^. Consistent with these previous observations, the ineffectiveness of memantine in enhancing SGZ cell proliferation in sedentary *dexras1*^*−/−*^ mice (Fig. 5K–M) suggests that NMDAR-mediated signaling is already suppressed in these animals, possibly due to uncoupling of the activated receptor from Dexras1-targeted effectors. In addition, Dexras1 is both a positive and a negative regulator of MAPK/ERK signaling, depending on cellular context and the nature of the stimulus^31,47,55,56^. For instance, light, acting through glutamate, triggers less ERK activation in the circadian clock of *dexras1*^*−/−*^ mice but, acting through the neuropeptide pituitary adenylate cyclase-activating peptide (PACAP), provokes greater ERK activity in these animals^45,57^. In the present study, running as a stimulus had no effect on ERK activity in the mutant DG compared to sedentary conditions.

In our hypothetical model (Fig. 6), Dexras1 serves to couple exercise-dependent NMDA receptor activity to ERK and CREB activation, and the expression of pro-mitogenic CREB target genes (e.g., *Bdnf*, *TrkB*), in the DG. In the absence of Dexras1, the activity or expression of these factors do not change in response to exercise (Fig. 5). Previous studies have suggested that wheel exercise activates hippocampal NMDA receptors, promoting BDNF expression and neurogenesis^58,59^. The proposed mechanism may be operating in mature DG granule neurons, which express high levels of Dexras1, NR2A, and BDNF and moderate levels of nNOS^54,60–62^. Under exercise conditions in wild-type mice, increased production of BDNF by granule neurons may enhance paracrine activation of TrkB receptors on type-1 cells, inducing their recruitment into the cell cycle by triggering CREB-dependent transcription of G1 cyclins and/or phosphorylation-induced degradation of cyclin-dependent kinase inhibitors^63–67^. The pro-mitogenic effects of exercise may be sustained in wild-type mice by a positive feedback mechanism that bolsters NMDA receptor activity in DG granule neurons through upregulation of NR2A, either at the transcriptional (e.g., by a BDNF-CREB pathway) or post-translational level (e.g., BDNF-induced phosphorylation or plasma membrane trafficking of NR2A subunits)^68–70^. In our mutant model, basal elevation of NR2A and CREB may alter this feedback mechanism by increasing the stimulus threshold needed to elicit a response in the quiescent and mitotic cell populations in the SGZ. It is also worth noting that Dexras1 may have additional roles within type-1 cells that are not captured in this model.

Dexras1 may regulate the exercise-mediated expansion of the proliferative pool via the above-mentioned mechanism, but this mechanism does not explain the observed differences in cell survival throughout neurogenesis in *dexras1*^*−/−*^ mice. Our results, as well as previous cell-fate experiments in sedentary wild-type mice, suggest that cell death in the hippocampal neurogenic niche begins as early as the first 1–2 days of a newborn cell’s life, and continues until mature neurons are synaptically integrated^34–36^. Studies using *bcl-2* over-expressing mice and *bax* knockout mice showed that apoptosis is the main driving force for the elimination of post-mitotic cells during adult neurogenesis^9,33^. Based on these studies, cell death at the mitotic stages, if it were to occur, would operate through an apoptosis-independent mechanism. Interestingly, Gascon *et al*. recently showed that ferroptosis—a programmed form of cell death characterized by iron-dependent accumulation of lipid-based reactive oxygen species—may play a role in the death of cells that fail to undergo neuronal conversion during somatic cell reprogramming^71^. Furthermore, exercise has been shown to reduce oxidative stress in the hippocampus^72,73^. It is possible that this reduction in oxidative stress inhibits ferroptosis in proliferating neural progenitor cells in wild-type mice. Of note, a recent study by Pilz *et al*., using chronic *in vivo* imaging in mice, demonstrated the occurrence of two distinct, critical periods of cell loss throughout the neurogenic program^74^. They propose that cell loss initially occurs within the first few days following cell cycle entrance, during the proliferative stages, and the second wave of cell clearance occurs approximately 1 to 3 weeks after new neurons are born, during the maturation stage^74^. This model is in line with the findings of our present study.

In *dexras1*^*−/−*^ mice, cell survival was altered at multiple neurogenic stages, suggesting that Dexras1 may control cell death by more than one mechanism. The potential existence of two cell death mechanisms, ferroptosis and apoptosis, in the hippocampus may explain why *dexras1* ablation has both pro- and anti-cell survival effects in this neurogenic niche. NMDA receptor activation has been shown to stimulate cellular uptake of iron through the iron channel divalent metal transporter 1 (DMT1), in a manner dependent on nitrosylated Dexras1^27,30,46^. It is possible that the enhanced survival of early-dividing cells in exercised *dexras1*^*−/−*^ mice may be due to suppressed NMDAR-dependent iron uptake in the hippocampi of these animals, reducing levels of ferroptotic cell death. At the post-mitotic stages, Dexras1 may be working through different mechanisms to promote or suppress apoptosis. Previous studies showed that Dexras1 over-expression increased the rate of apoptosis in cancer cell lines, an effect that was dependent on the GTP/GDP binding activity of Dexras1^75^. Interestingly, Dexras1 can inhibit signaling downstream of dopamine D1 receptors, whose activation has been shown to promote neuroblast survival^76,77^. These mechanisms are highly speculative and necessitate new methods to better study cell turnover in the hippocampus. Regardless of the underlying mechanisms, our study identifies Dexras1 as an important stage-specific regulator of exercise-dependent hippocampal neurogenesis through its effects on cell proliferation and survival.

## Materials and Methods

### Animals and Ethics Statement

*Dexras1*^+/−^ mice were backcrossed for at least 13 generations onto a C57BL/6 J background prior to establishment of a *dexras1*^*−/−*^ colony. C57BL/6 J mice were purchased from Jackson Laboratories (Bar Harbor, ME, USA), bred in-house, and used as wild-type controls. Mice were 35- to 40-days-old at the start of each experiment^45^. All animal handling and experimental procedures were performed at the University of Toronto Mississauga (UTM) Animal Facility and were approved by the UTM Animal Care Committee, complying with guidelines established by the University of Toronto Animal Care Committee and the Canadian Council on Animal Care.

### Thymidine analog and memantine injections

For all thymidine analogue injection paradigms, mice were singly housed in polycarbonate cages (33 × 15 × 13 cm) with *ad libitum* access to rodent chow and water throughout the experiment. Cages were either equipped with a red dome for shelter (sedentary) or with a running wheel (10.8 cm diameter) (exercise) and placed in light-tight ventilated cabinets under a 12-h light:12-h dark (LD) cycle for the duration of the experiments. For thymidine analog-injection paradigms, mice received equimolar intraperitoneal (i.p.) injection(s) of either 5-bromo-2′-deoxyuridine (BrdU, Sigma-Aldrich, St. Louis, MO, USA) in 0.9% NaCl solution at a dose of 100 mg/kg, 5-iodo-2′-deoxyuridine (IdU, Sigma-Aldrich) in 0.9% NaCl solution at a dose of 57.5 mg/kg, or 5-chloro-2′-deoxyuridine (CldU, Sigma-Aldrich) in 0.9% NaCl solution at a dose of 42.5 mg/kg. Cell cycle kinetics analysis was performed as previously described with modifications^42,78^. To quantify the number of S-phase cells, mice were injected once on day 5 of exercise condition and tissues were harvested 1 hr post-injection (Fig. 1). For the BrdU-label retaining experiment (Fig. 1), mice were injected once-daily with BrdU from days 1 to 5 of exercise condition, and tissues were harvested 28 days after the first injection. For the fate-tracing experiment (Fig. 2), mice were injected once with BrdU on day 5 of exercise condition and tissues were harvested after 1 hr or after 1, 5, 14, or 28 days post-injection. To analyse cell cycle entrance and exit (Fig. 3), mice were injected once with BrdU on day 5 of exercise condition. For cell cycle entrance analysis, tissues were harvested 1 hr post-injection. For cell cycle exit, tissues were harvested 24 hr post-injection. For cell cycle kinetics calculations (Fig. 4), mice were injected once with IdU on day 5 of exercise, followed by a single CldU injection at either 4 hr or 18 hr post-IdU injection for S-phase or cell cycle length calculation, respectively. Tissues were harvested 45 min post-CldU injection. For memantine experiments (Fig. 5), 35-day-old mice received a single intraperitoneal injection of memantine hydrochloride (Tocris Bioscience, Bristol, UK) diluted in DMSO:0.9% NaCl (1:1 v:v) at a dose of 50 mg/kg. After 48 hr, mice received three BrdU injections spaced 2 hr apart, and tissues were harvested 2 hr after the last BrdU injection.

### Tissue processing

Tissue processing was previously described in Bouchard-Cannon *et al*.^42^. Mice were killed by cervical dislocation, and brains were sectioned in cooled oxygenated media using an oscillating tissue slicer to obtain an 800-μm thick coronal section containing the medial hippocampus. Tissues were quickly placed in 4% (w/v) paraformaldehyde in phosphate-buffered saline (PBS, pH 7.4) and fixed for 6 hr at room temperature. Tissues were then transferred to 30% (w/v) sucrose in PBS (overnight, 4 °C), and subsequently processed to 30-μm thin sections using a freezing microtome. For protein or RNA extraction, brains were sectioned by vibratome into two 400-μm thick coronal sections in either oxygenated media or ice-cold DEPC-treated PBS, respectively. The DG was micro-dissected under a stereomicroscope using a syringe needle. Samples were frozen on dry ice and stored at −80 °C until further processing.

### Immunohistochemistry (IHC) and co-immunofluorescence (co-IF)

For BrdU immunohistochemistry (IHC) and co-immunofluorescence (co-IF), tissues were first treated with 2N HCl for 30 min at 37 °C in a hybridizing incubator, washed once in PBS (pH 7.4), and quenched with 0.05M borate buffer (pH 8.3) for 30 min at room temperature (RT) or 0.1M borate buffer (pH 8.5) for 20 min at RT for IdU/CldU staining. Sections were washed five times with PBS before continuing with regular IHC and co-IF protocols.

For IHC, sections were treated with 0.3% H_2_O_2_ in PBS for 20 min at RT, washed with PBS, blocked with 10% horse serum in PBS containing 0.1% Triton X-100 (PBS-T) for 1 hr, and incubated overnight with primary antibodies diluted in blocking solution. Sections were subsequently washed with PBS-T and incubated with horseradish peroxidase (HRP)-conjugated secondary antibodies for 2 hr at RT. After PBS-T washes, sections were incubated for 45 min in Vectastain ABC Reagent (Vector Laboratories, Burlington, ON, Canada) and developed with nickel-intensified 3,3′-diaminobenzidine (DAB; Vector Laboratories). Sections were mounted on gelatin-coated microscope slides, dehydrated, and cover-slipped with Permount Mounting Media (Fisher Scientific, Ottawa, ON, Canada).

For all co-IF that do not include BrdU, sections were blocked for 1 hr, incubated overnight in primary antibodies at 4 °C, washed with PBS-T, and incubated at RT for 2 hr in secondary antibodies protected from light. For co-IF with BrdU, sections were incubated overnight with the BrdU primary antibody, washed with PBS-T, incubated for a second overnight period with remaining primary antibodies, washed with PBS-T, and incubated in all secondary antibodies on the following day. For IdU/CldU staining, sections were incubated in a stringent blocking buffer containing 50 mM glycine, 10% horse serum, 2% BSA and 0.3% Triton-X100 in PBS. Sections were mounted on microscope slides, cover-slipped with Fluorescence Mounting Media (Dako Canada, Inc., Burlington, ON, Canada), sealed with nail-polish and stored at 4 °C. See Table S3 for antibody concentrations.

### Imaging

Images were acquired using a Zeiss Axio Oserver Z1 inverted microscope equipped with a Laser Scanning Microscope (LSM) 700 module (Zeiss, Oberkochen, Germany) for confocal images, and an AxioCam MRm Rev.3 monochromatic digital camera (Zeiss) for bright-field pictures, and operated with the Zen 2010 software (Zeiss). IHC and IF images were acquired using 10× or 40× objectives, respectively. For IF, fluorochrome signals were collected serially, with barrier filters manually set. Unless indicated otherwise, all images correspond to a single z-stack of 2-μm focal plane orthogonal projections. Identical confocal imaging settings were used within experiments.

### Quantification

Quantification was performed using the ImageJ software (http://rsbweb.nih.gov/ij). The number of cells per μm^3^ of SGZ (12-μm thickness), 2× SGZ (24-μm thickness) or GCL (manually selected) was measured by dividing the total number of cells within the region of interest by the tissue volume (area x 30-μm) (Fig. S4). The percentage of type-1 cells entering the cell cycle was calculated by dividing the number of SOX2^+^GFAP^+^BrdU^+^ cells by the total number of SOX2^+^GFAP^+^ cells from four to six coronal hippocampal sections per animal. The percentage of proliferating cells that have exited the cell cycle after 24 hr was determined by dividing the number of BrdU^+^Ki-67^−^ cells by the total number of BrdU^+^ cells within the SGZ of six coronal hippocampal sections per animal. For S-phase length (T_s_) calculations, the total number of IdU^+^ cells was multiplied by the inter-injection interval (4 hr) and divided by the number of IdU^+^CldU^-^ cells within the SGZ. For cell cycle length (T_c_) calculations, the previously calculated mean S-phase length (T_s_) was multiplied by the number of CldU^+^IdU^-^ cells and divided by the total number of CldU^+^ cells. This value was added to the inter-injection interval (18 hr). The combined G1/G2/M-phase length was estimated by subtracting the mean S-phase length (T_s_) from the mean cell cycle length (T_c_)^78^. To estimate the percentage of dividing cells that entered a second cell cycle within 18 hours, we used the injection paradigm for T_c_ calculations, dividing the number of IdU^+^CldU^+^ cells by the total number of IdU^+^ cells and multiplying that value by 100.

### RNA extraction and qPCR

Total RNA was isolated from micro-dissected DG tissues using TRIzol Reagent (Invitrogen-Thermo Scientific, Burlington, ON, Canada). cDNA synthesis was performed using SuperScript IV Reverse Transcriptase (Invitrogen). RT-PCR was performed using the SsoFast EvaGreen Supermix with low ROX (Bio-Rad, Mississauga, ON, CA) along with primer sets indicated in Table S4^54,79–81^. Values were normalized to *gapdh* abundance.

### Western blotting

Micro-dissected DG tissues were homogenized on ice in RIPA buffer (10 mM Tris-Cl pH 8.0, 1 mM EDTA, 1% Triton X-100, 0.1% sodium deoxycholate, 0.1% SDS, 140 mM NaCl, 1 mM β-glycerophosphate, 1 mM sodium orthovanadate, 5 mM sodium fluoride, Protease Inhibitor Cocktail [Sigma-Aldrich]) using disposable pellet pestles. Sample concentration was determined using the Bradford method. Samples were diluted in RIPA buffer to a final concentration of 2 ug/uL in 1× loading buffer (50 mM Tris-CL pH6.8, 2% SDS, 10% glycerol, 1% β-mercaptoethanol, 12.5 mM EDTA, 5 mM DTT, 0.02% bromophenol blue). Samples were resolved on an 8% Tris-glycine SDS-PAGE gel and blotted by wet transfer onto PVDF membranes (Immobilon-P; Thermo Fisher Scientific Inc.). Membranes were washed with TBS-T (20 mM Tris base, 150 mM NaCl, 0.05% Tween-20), blocked with 5% skim milk in TBS-T, and incubated overnight at 4 °C with primary antibodies. Membranes were washed and incubated with secondary antibodies for 2 hr at RT. Band signals were detected by chemiluminescence using the SuperSignal West Femto Maximum Sensitivity Substrate (Thermo Fischer Scientific), exposed on film and developed for optimal visualization. Band intensity was measured using the ImageJ software (http://rsbweb.nih.gov/ij). Antibody concentrations can be found in Table S3.

### Statistical analysis

Statistics were performed using statistical tools from Minitab® software version 17.1.0 (Minitab Inc., State College, PA, USA). Data were analyzed using one- or two-way ANOVA of independent and repeated measures, followed by post-hoc Fisher’s LSD tests with alpha set at 0.05.

## Electronic supplementary material

Supplementary Information

## References

[1] Stolp HB, Molnár Z (2015). Neurogenic niches in the brain: help and hindrance of the barrier systems. Front. Neurosci..

[2] Seri B, Garcia-Verdugo JM, Mcewen BS, Alvarez-buylla A (2001). Astrocytes Give Rise to New Neurons in the Adult Mammalian Hippocampus. J. Neurosci..

[3] Babu H, Cheung G, Kettenmann H, Palmer TD, Kempermann G (2007). Enriched Monolayer Precursor Cell Cultures from Micro- Dissected Adult Mouse Dentate Gyrus Yield Functional Granule Cell-Like Neurons. PLoS One.

[4] Kempermann G, Song H, Gage FH (2015). Neurogenesis in the AdultHippocampus. Cold Spring Harb. Perspect. Biol..

[5] Steiner B (2006). Type-2 Cells as Link Between Glial and Neuronal Lineage in Adult Hippocampal Neurogenesis. Glia.

[6] Brown JP (2003). Transient Expression of Doublecortin during Adult Neurogenesis. J. Comp. Neurol..

[7] Jessberger S, Römer B, Babu H, Kempermann G (2005). Seizures induce proliferation and dispersion of doublecortin-positive hippocampal progenitor cells. Exp. Neurol..

[8] Toni N, Schinder AF (2016). Maturation and Functional Integration of New Granule Cells into the AdultHippocampus. Cold Spring Harb. Perspect. Biol..

[9] Kuhn HG, Biebl M, Wilhelm D, Li M, Friedlander RM (2005). Increased generation of granule cells in adult Bcl-2- overexpressing mice: a role for cell death during continued hippocampal neurogenesis. Eur. J. Neurosci..

[10] Li Y, Cheng Z, Wong S (2016). Differential Apoptosis Radiosensitivity of Neural Progenitors in Adult Mouse Hippocampus. Int. J. Mol. Sci..

[11] Encinas JM, Sierra A (2012). Neural stem cell deforestation as the main force driving the age-related decline in adult hippocampal neurogenesis. Behav. Brain Res..

[12] Lugert S (2010). Quiescent and active hippocampal neural stem cells with distinct morphologies respond selectively to physiological and pathological stimuli and aging. Cell Stem Cell.

[13] Luo Y (2010). Fragile X mental retardation protein regulates proliferation and differentiation of adult neural stem/progenitor cells. PLoS Genet..

[14] Monteiro BMM, Moreira FA, Massensini AR, Moraes MFD, Pereira GS (2014). Enriched environment increases neurogenesis and improves social memory persistence in socially isolated adult mice. Hippocampus.

[15] Dranovsky A (2011). Experience Dictates Stem Cell Fate in the Adult Hippocampus. Neuron.

[16] van Praag H, Christie BR, Sejnowski TJ, Gage FH (1999). Running enhances neurogenesis, learning, and long-term potentiation in mice. Proc Natl Acad Sci USA.

[17] Vilar M, Mira H (2016). Regulation of neurogenesis by neurotrophins during adulthood: Expected and unexpected roles. Front. Neurosci..

[18] Berg DA, Belnoue L, Song H, Simon A (2013). Neurotransmitter-mediated control of neurogenesis in the adult vertebrate brain. Development.

[19] Piatti VC (2011). The timing for neuronal maturation in the adult hippocampus is modulated by local network activity. J. Neurosci..

[20] Hsieh J, Zhao X (2016). Genetics and Epigenetics in AdultNeurogenesis. Cold Spring Harb. Perspect. Biol..

[21] Cismowski MJ (2000). Activation of Heterotrimeric G-protein Signaling by a Ras-related Protein. J. Biol. Chem..

[22] Graham TE, Prossnitz ER, Dorin RI (2002). Dexras1/AGS-1 inhibits signal transduction from the Gi-coupled formyl peptide receptor to Erk-1/2 MAP kinases. J. Biol. Chem..

[23] Graham TE, Qiao Z, Dorin RI (2004). Dexras1 inhibits adenylyl cyclase. Biochem. Biophys. Res. Commun..

[24] Takesono A, Nowak MW, Cismowski M, Duzic E, Lanier SM (2002). Activator of G-protein signaling 1 blocks GIRK channel activation by a G-protein-coupled receptor. Apparent disruption of receptor signaling complexes. J. Biol. Chem..

[25] Cismowski MJ (1999). Genetic screens in yeast to identify mammalian nonreceptor modulators of G-protein signaling. Nat. Biotechnol..

[26] Fang M (2000). Dexras1: A G Protein Specifically Coupled to Neuronal Nitric Oxide Synthase via CAPON. Neuron.

[27] Cheah JH (2006). NMDA receptor-nitric oxide transmission mediates neuronal iron homeostasis via the GTPase Dexras1. Neuron.

[28] Jaffrey SR, Fang M, Snyder SH (2002). Nitrosopeptide Mapping: A Novel Methodology Reveals S -Nitrosylation of Dexras1 on a Single Cysteine Residue. Chem. Biol..

[29] Chen Y, Mathias L, Falero-perez JM, Kim SF (2015). PKA-mediated phosphorylation of Dexras1 suppresses iron trafficking by inhibiting S-nitrosylation. Fed. Eur. Biochem. Soc..

[30] White RS (2016). Lysosomal iron modulates NMDA receptor-mediated excitation via small GTPase, Dexras1. Mol. Brain.

[31] Kim HJ (2016). Dexras1 links glucocorticoids to insulin-like growth factor-1 signaling in adipogenesis. Sci. Rep..

[32] Bouchard-Cannon P, Cheng H-YM (2012). Scheduled Feeding Alters the Timing of the Suprachiasmatic Nucleus Circadian Clock in Dexras1-Deficient Mice. Chronobiol. Int..

[33] Sun W (2004). Programmed Cell Death of Adult-Generated Hippocampal Neurons Is Mediated by the Proapoptotic Gene Bax. J. Neurosci..

[34] Encinas JM (2011). Division-coupled astrocytic differenciation and age-related depletion of neural stem cells in the adult hippocampus. Cell Stem Cell.

[35] Sierra A (2015). Neuronal hyperactivity accelerates depletion of neural stem cells and impairs hippocampal neurogenesis. Cell Stem Cell.

[36] Mandyam CD, Harburg GC, Eisch AJ (2007). Determination of key aspects of precursor cell poliferation, cell cycle length and kinetics in the adult mouse subgranular zone. Neuroscience.

[37] Overall RW, Walker TL, Fischer TJ, Brandt MD, Kempermann G (2016). Different mechanisms must be considered to explain the increase in hippocampal neural precursor cell proliferation by physical activity. Front. Neurosci..

[38] Sierra A (2010). Article Microglia Shape Adult Hippocampal Neurogenesis through Apoptosis-Coupled Phagocytosis. Cell Stem Cell.

[39] Gemma C, Bachstetter AD (2013). The role of microglia in adult hippocampal neurogenesis. Front. Cell. Neurosci..

[40] Lu Z (2011). Phagocytic activity of neuronal progenitors regulates adult neurogenesis. Nat. Cell Biol..

[41] Brandt MD, Maass A, Kempermann G, Storch A (2010). Physical exercise increases Notch activity, proliferation and cell cycle exit of type-3 progenitor cells in adult hippocampal neurogenesis. Mol. Dev. Neurosci..

[42] Bouchard-Cannon P, Mendoza-Viveros L, Yuen A, Kærn M, Cheng H-YM (2013). The Circadian Molecular Clock Regulates Adult Hippocampal Neurogenesis by Controlling the Timing of Cell-Cycle Entry and Exit. Cell Rep..

[43] Farhy C (2013). Pax6 Is Required for Normal Cell-Cycle Exit and the Differentiation Kinetics of Retinal Progenitor Cells. PLoS One.

[44] Chen MJ, Russo-Neustadt AA (2009). Running exercise-induced up-regulation of hippocampal brain-derived neurotrophic factor is CREB-dependent. Hippocampus.

[45] Cheng H-YM (2004). Dexras1 potentiates photic and suppresses nonphotic responses of the circadian clock. Neuron.

[46] Chen Y (2013). Dexras1, a Small GTPase, Is Required for Glutamate-NMDA Neurotoxicity. J. Neurosci..

[47] Zhu L-J (2014). CAPON-nNOS coupling can serve as a target for developing new anxiolytics. Nat. Med..

[48] Carlson GC (2016). Dexras1 A UNIQUE ras-GTPase Interacts With Nmda Receptor Activity and Provides A Novel Dissociation Between Anxiety, Working Memory and Sensory Gating. Neuroscience.

[49] Maekawa M (2009). NMDA receptor antagonist memantine promotes cell proliferation and production of mature granule neurons in the adult hippocampus. Neurosci. Res..

[50] Kronenberg G (2003). Subpopulations of Proliferating Cells of the Adult Hippocampus Respond Differently to Physiologic Neurogenic Stimuli. J. Comp. Neurol..

[51] Steiner B, Zurborg S, Fabel K, Kempermann G (2008). Differential 24 H Responsiveness of Prox1– Expressing Precursor Cells In Adult Hippocampal Neurogenesis to Physical Activity, Environmental Enrichment, and Kainic Acid–Induced Seizures. Neuroscience.

[52] Brandt MD (2003). Transient calretinin expression defines early postmitotic step of neuronal differentiation in adult hippocampal neurogenesis of mice. Mol. Cell. Neurosci..

[53] Fischer TJ, Walker TL, Overall RW, Brandt MD, Kempermann G (2014). Acute effects of wheel running on adult hippocampal precursor cells in mice are not caused by changes in cell cycle length or S phase length. Front. Neurosci..

[54] Takahashi H (2003). Mouse dexamethasone-induced RAS protein 1 gene is expressed in a circadian rhythmic manner in the suprachiasmatic nucleus. Mol. Brain Res..

[55] Lellis-santos C (2018). The Regulation of Rasd1 Expression by Glucocorticoids and Prolactin Controls Peripartum Maternal Insulin Secretion. Gen. Endocrinol..

[56] Cha JY (2013). Dexras1 mediates glucocorticoid-associated adipogenesis and diet-induced obesity. Proc. Natl. Acad. Sci. USA.

[57] Cheng H-YM (2006). The molecular gatekeeper Dexras1 sculpts the photic responsiveness of the mammalian circadian clock. J. Neurosci..

[58] Kitamura T, Mishina M, Sugiyama H (2003). Enhancement of neurogenesis by running wheel exercises is suppressed in mice lacking NMDA receptor o 1 subunit. Neurosci. Res..

[59] Mattson MP (2008). Glutamate and Neurotrophic Factors in Neuronal Plasticity and Disease. Ann. N. Y. Acad. Sci..

[60] Wenzel A, Fritschy JM, Mohier H, Benke D (1997). NMDA Receptor Heterogeneity During Postnatal Development of the Rat Brain: Differential Expression of the NR2A, NR2B, and NR2C Subunit Proteins. J. Neurochem..

[61] Burette A, Zabel U, Weinberg RJ, Schmidt HHHW, Valtschanoff JG (2002). Synaptic Localization of Nitric Oxide Synthase and Soluble Guanylyl Cyclase in the Hippocampus. J. Neurosci..

[62] Renouard L (2015). The supramammillary nucleus and the claustrum activate the cortex during REM sleep. Neurophysiology.

[63] Benraiss A, Chmielnicki E, Lerner K, Roh D, Goldman SA (2001). Adenoviral brain-derived neurotrophic factor induces both neostriatal and olfactory neuronal recruitment from endogenous progenitor cells in the adult forebrain. J. Neurosci..

[64] Kowalczyk A (2004). The critical role of cyclin D2 in adult neurogenesis. J. Cell Biol..

[65] White PC (2006). Regulation of cyclin D2 and the cyclin D2 promoter by protein kinase A and CREB in lymphocytes. Oncogene.

[66] Kuczewski N (2008). Backpropagating Action Potentials Trigger Dendritic Release of BDNF during Spontaneous Network Activity. J. Neurosci..

[67] Li N, Wang C, Wu Y, Liu X, Cao X (2009). Ca2+/calmodulin-dependent protein kinase II promotes cell cycle progression by directly activating MEK1 and subsequently modulating p27 phosphorylation. J. Biol. Chem..

[68] Levine ES, Crozier RA, Black IB, Plummer MR (1998). Brain-derived neurotrophic factor modulates hippocampal synaptic transmission by increasing N-methyl-D-aspartic acid receptor activity. Proc. Natl. Acad. Sci. USA.

[69] Caldeira MV (2007). BDNF regulates the expression and traffic of NMDA receptors in cultured hippocampal neurons. Mol. Cell. Neurosci..

[70] Murray PS, Holmes PV (2011). An Overview of Brain-Derived Neurotrophic Factor and Implications for Excitotoxic Vulnerability in theHippocampus. Int. J. Pept..

[71] Gascon S (2016). Identification and Successful Negotiation of a Metabolic Checkpoint in Direct Neuronal Identification and Successful Negotiation of a Metabolic Checkpoint in Direct Neuronal Reprogramming. Cell Stem Cell.

[72] Salim S (2011). Potential contribution of oxidative stress and inflammation to anxiety and hypertension. Brain Res..

[73] Marosi K (2012). Long-Term Exercise Treatment Reduces Oxidative Stress In the Hippocampus of Aging Rats. Neuroscience.

[74] Pilz G (2018). Live imaging of neurogenesis in the adult mouse hippocampus. Science (80−.)..

[75] Vaidyanathan G (2004). The Ras-related protein AGS1/RASD1 suppresses cell growth. Oncogene.

[76] Harrison LM, He Y (2011). Rhes and AGS1/Dexras1 Affect Signaling by Dopamine D1 Receptors Through Adenylyl Cyclase. J. Neurosci. Res..

[77] Takamura N (2014). The effect of dopamine on adult hippocampal neurogenesis. Prog. Neuro-Psychopharmacology Biol. Psychiatry.

[78] Brandt MD, Hübner M, Storch A (2012). Adult Hippocampal Precursor Cells Shorten S-Phase and Total Cell Cycle Length During Neuronal Differentiation. Stem Cells.

[79] Son Y (2015). Cranial irradiation regulates CREB-BDNF signaling and variant BDNF transcript levels in the mouse hippocampus. Neurobiol. Learn. Mem..

[80] Guo X (2014). PGC-1α signaling coordinates susceptibility to metabolic and oxidative injury in the inner retina. Am. J. Pathol..

[81] Bick-Sander A, Steiner B, Wolf SA, Babu H, Kempermann G (2006). Running in pregnancy transiently increases postnatal hippocampal neurogenesis in the offspring. Proc. Natl. Acad. Sci. USA.

