# Supplementary material for: Dexras1 is a homeostatic regulator of exercise-dependent proliferation and cell survival in the hippocampal neurogenic niche
Source: Sci Rep. 2018 Mar 28;8:5294. doi: 10.1038/s41598-018-23673-z (PMC5871767; doi:10.1038/s41598-018-23673-z)
Supplement: Supplementary file 1 — Supplementary Information [file 41598_2018_23673_MOESM1_ESM.pdf]

Supplemental Information

Dexras1 is a homeostatic regulator of exercise-dependent proliferation and cell survival in the hippocampal neurogenic niche

Pascale Bouchard-Cannon,[1,2] Christopher Lowden[1,2], Dennison Trinh, [1,2] Hai-Ying M. Cheng[1,2*]

Supplemental Figures

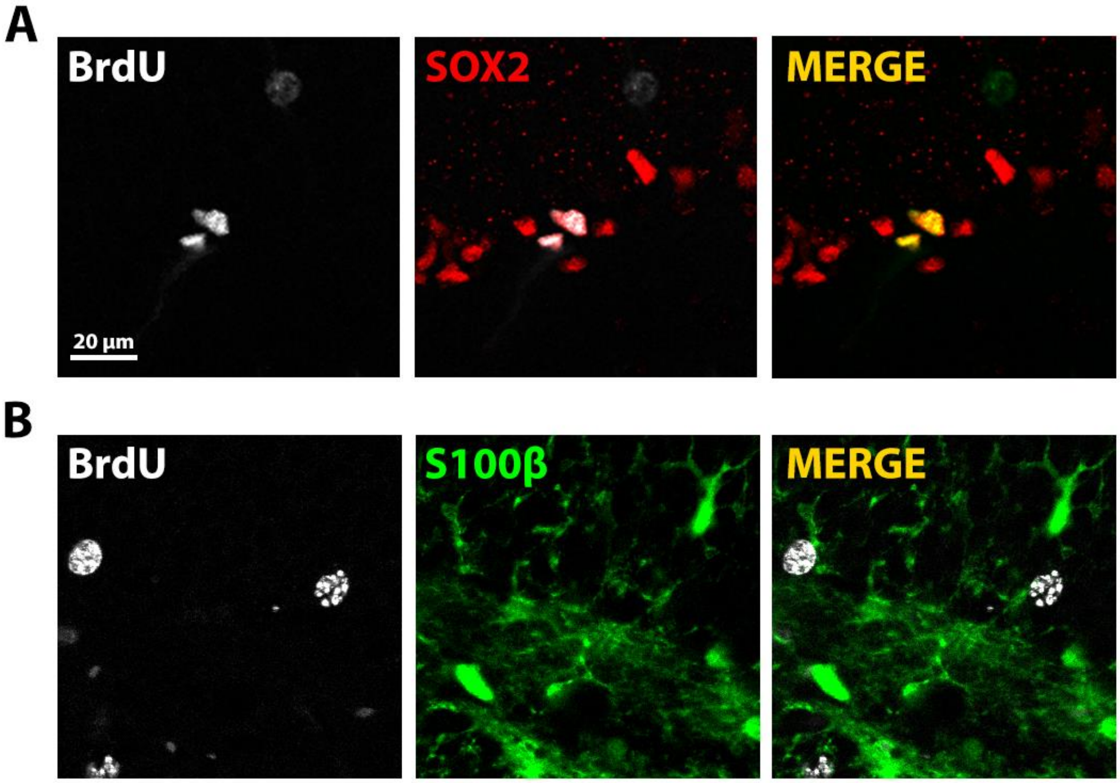

Figure S1. Co-localization of 28-days post-injection (DPI) label-retaining BrdU$^+$ cells with SOX2 or S100β within the Subgranular Zone (SGZ) and Dentate Gyrus (DG). (A) Representative photomicrograph of BrdU (white) and SOX2 (red) co-localization in the SGZ at 28 DPI. **(B)** Representative photomicrograph of BrdU (white) and S100B (green) expression in the DG at 28 DPI.

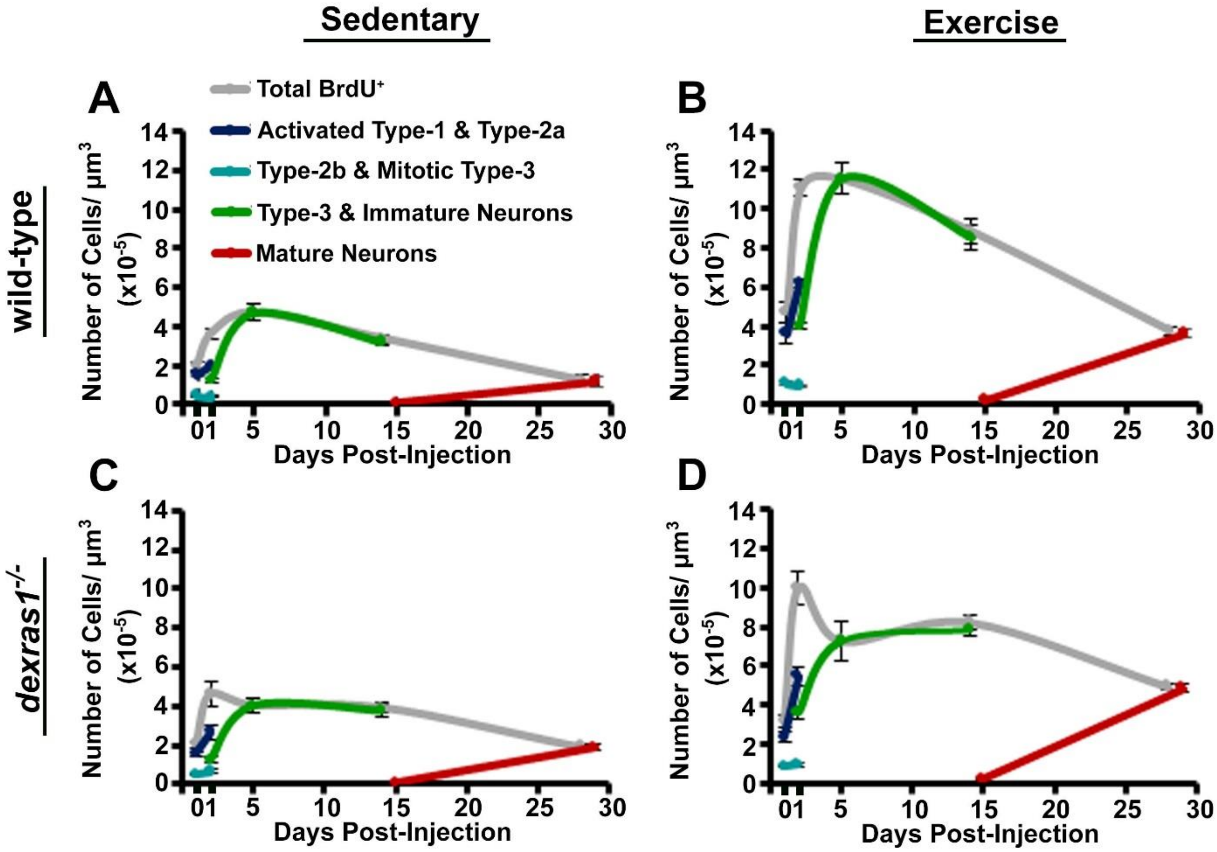

Figure S2. Fate of BrdU-labeled dividing cells in the SGZ of sedentary and exercised mice. (A-D) Quantification of the number of total BrdU$^+$ cells (grey), activated type-1 & type-2a cells (blue), type-2b & mitotic type-3 cells (turquoise), post-mitotic type-3 & immature neurons (green), and mature neurons (red) per µm^3 of SGZ (x10^{-5}) from 1 hour to 28 days post-BrdU injection, in **(A)** sedentary wild-type mice, **(B)** exercised wild-type mice, **(C)** sedentary *dexras1*$^{-/-}$ mice, and **(D)** exercised *dexras1*$^{-/-}$ mice. All values represent mean ± standard error.

A

$$\%\ \text{Cell cycle re-entrance} = 100 \times \frac{(CldU^{+}IdU^{+})}{IdU^{+}}$$

B

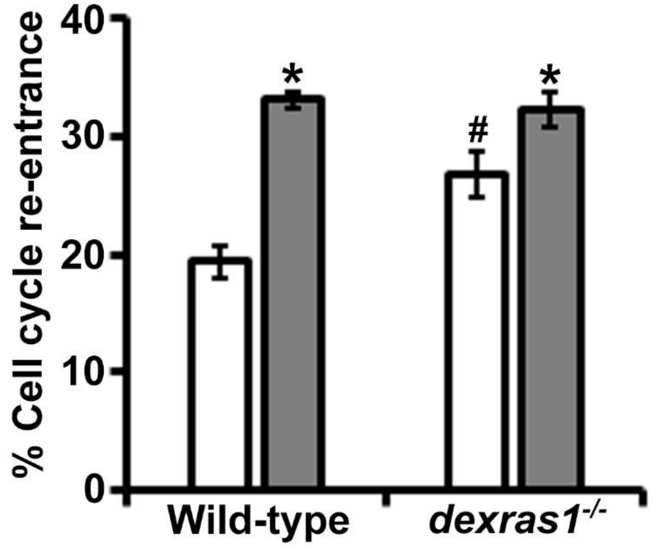

Figure S3. Exercise enhances cell cycle re-entrance of type-2 cells. Relates to Fig. 4. Wild-type and *dexras1*$^{-/-}$ mice were injected with equimolar amounts of IdU and CldU, spaced 18-hrs apart, after either 5 days of sedentary or exercise conditions. **(A)** Formula used to calculate the estimated % cell cycle re-entrance of S-phase cells after 18-hrs. **(B)** Quantification of the % cell cycle entrance. Values represent mean ± standard error or estimated value. *p<0.05 vs. sedentary control. $^{\#}p$<0.05 vs. wild-type control. n= 4-5 per group.

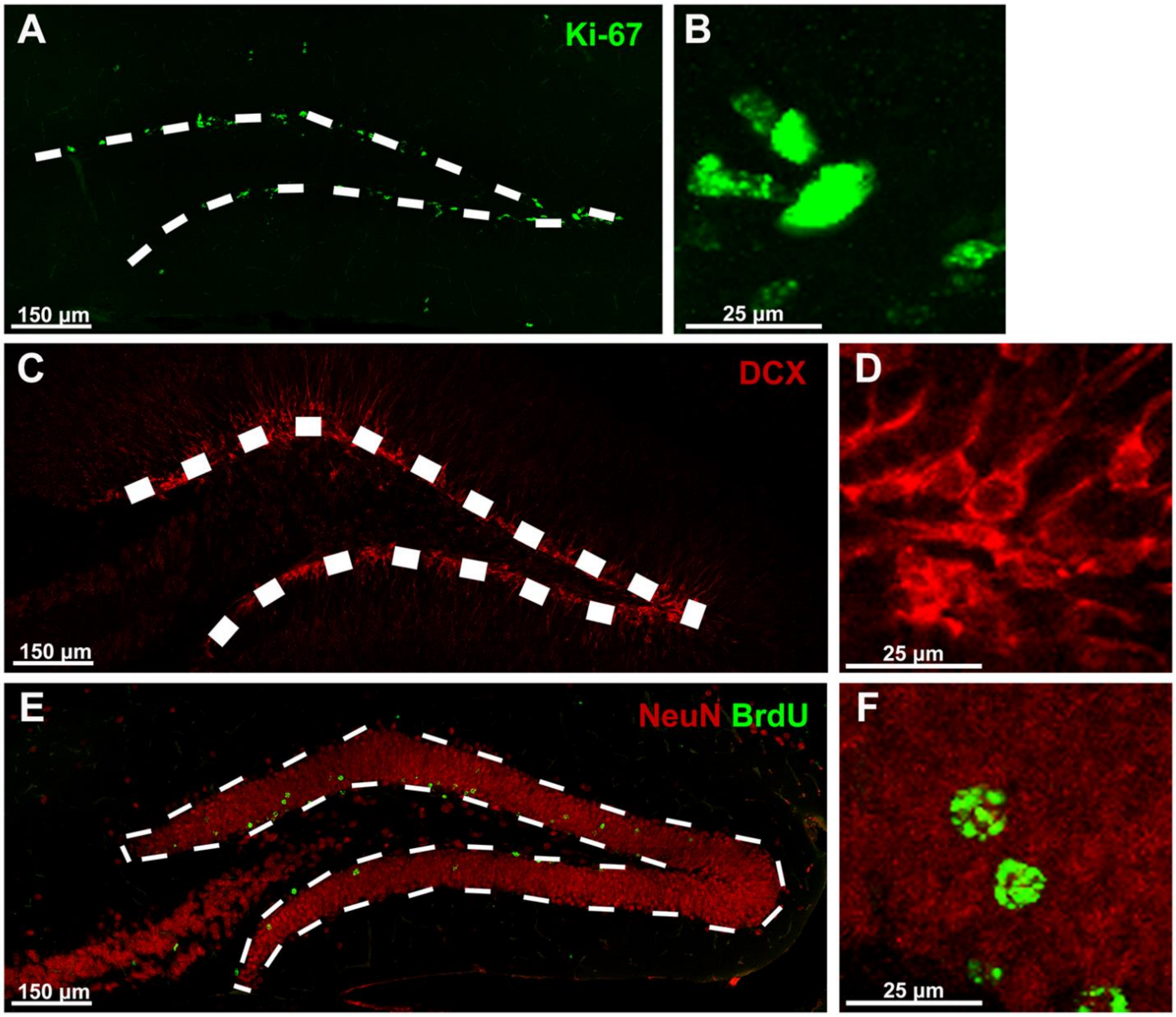

Figure S4. Delineated hippocampal area used for density measurements (A, C, E) Representative photomicrographs of **(A)** Ki-67, **(C)** DCX, and **(E)** NeuN/ BrdU. White hashed lines represent the area used for density measurements. (**B, D, F)** Zoomed-in images of **(B)** Ki-67, **(D)** DCX, and **(F)** NeuN/ BrdU. Images are represented as a **(A-B, E-F)** collapsed z-stack image (30-µm) or **(C-D)** a single z-stack image (5-µm) acquired at 40x magnification.

Supplemental Tables

Table S1. Label Retaining Assay Cell Counts

Total BrdU (number of cells per um^3) (x10^{-5})						
Genotype	Condition	1 HPI	1 DPI	5 DPI	14 DPI	28 DPI
wild-type	Sedentary	2.0 ± 0.2	3.6 ± 0.3	5.3 ± 0.5	3.4 ± 0.2	1.3 ± 0.3
wild-type	Exercise	4.7 ± 0.6*	11.1 ± 0.4*	12.2 ± 0.8*	8.9 ± 0.6*	2.3 ± 0.2
dexras1$^{-/-}$	Sedentary	2.0 ± 0.1	4.6 ± 0.6	4.4 ± 0.4	3.9 ± 0.2	1.9 ± 0.1
dexras1$^{-/-}$	Exercise	3.1 ± 0.3*#	10.0 ± 0.8*	7.8 ± 0.1*#	8.2 ± 0.4*	4.9 ± 0.2*#
**Total Type 2a (BrdU$^+$ Ki-67$^+$ DCX$^-$) (number of cells per um^3) (x10^{-5})**						
Genotype	Condition	1 HPI	1 DPI	5 DPI	14 DPI	28 DPI
wild-type	Sedentary	1.5 ± 0.1	2.0 ± 0.2	ND	ND	ND
wild-type	Exercise	3.6 ± 0.5*	6.1 ± 0.2*	ND	ND	ND
dexras1$^{-/-}$	Sedentary	1.5 ± 0.9	2.7 ± 0.4	ND	ND	ND
dexras1$^{-/-}$	Exercise	2.3 ± 0.3*#	5.5 ± 0.5*	ND	ND	ND
**Total Type 2b (BrdU$^+$ Ki-67$^+$ DCX$^+$) (number of cells per um^3) (x10^{-6})**						
Genotype	Condition	1 HPI	1 DPI	5 DPI	14 DPI	28 DPI
wild-type	Sedentary	4.4 ± 0.8	4.0 ± 0.2	ND	ND	ND
wild-type	Exercise	10.8 ± 1.2	9.3 ± 0.6*	ND	ND	ND
dexras1$^{-/-}$	Sedentary	4.8 ± 0.6	6.8 ± 1.1#	ND	ND	ND
dexras1$^{-/-}$	Exercise	8.4 ± 0.7	9.1 ± 0.8*	ND	ND	ND
**Total Type 3 (BrdU$^+$ Ki-67$^-$ DCX$^+$) (number of cells per um^3) (x10^{-5})**						
Genotype	Condition	1 HPI	1 DPI	5 DPI	14 DPI	28 DPI
wild-type	Sedentary	ND	1.2 ± 0.1	4.7 ± 0.4	3.2 ± 0.2	ND
wild-type	Exercise	ND	4.0 ± 0.2*	11.5 ± 0.8*	8.5 ± 0.6*	ND
dexras1$^{-/-}$	Sedentary	ND	1.2 ± 0.2	4.0 ± 0.4	3.8 ± 0.3	ND
dexras1$^{-/-}$	Exercise	ND	3.6 ± 0.3*	7.3 ± 1.0*#	7.8 ± 0.4*	ND
**Newborn Neurons (BrdU$^+$ NeuN$^+$) (number of cells per um^3) (x10^{-5})**						
Genotype	Condition	1 HPI	1 DPI	5 DPI	14 DPI	28 DPI
wild-type	Sedentary	ND	ND	ND	0.10 ± 0.01	1.2 ± 0.3
wild-type	Exercise	ND	ND	ND	0.18 ± 0.02*	3.6 ± 0.2*
dexras1$^{-/-}$	Sedentary	ND	ND	ND	0.07 ± 0.02	1.9 ± 0.1
dexras1$^{-/-}$	Exercise	ND	ND	ND	0.20 ± 0.03*	4.8 ± 0.2*#

ND : Not detected

** : Significant at p ≤ 0.05 within the same genotype.*

# : Significant at p ≤ 0.05 between genotypes of the same condition.

Table S2. Fold changes in BrdU$^+$ cells relative to genotype, condition and time

Fold change in BrdU within the same harvest time and genotype ((Exercise – Sedentary) / Sedentary)						
**Genotype**	**Condition**	**1 HPI**	**1 DPI**	**5 DPI**	**14 DPI**	**28 DPI**
wild-type	Sedentary	0.00	0.00	0.00	0.00	0.00
wild-type	Exercise	1.38	2.06	1.32	1.62	1.99
dexras1$^{-/-}$	Sedentary	0.00	0.00	0.00	0.00	0.00
dexras1$^{-/-}$	Exercise	0.56	1.18	0.79	1.10	1.58
**Fold Change in BrdU relative to 1 HPI within the same genotype and condition ((DPI – 1 HPI)/1 HPI)**						
**Genotype**	**Condition**	**1 HPI**	**1 DPI**	**5 DPI**	**14 DPI**	**28 DPI**
wild-type	Sedentary	0.00	0.83	1.66	0.72	-0.37
wild-type	Exercise	0.00	1.35	1.60	0.89	-0.20
dexras1$^{-/-}$	Sedentary	0.00	1.26	1.15	0.93	-0.06
dexras1$^{-/-}$	Exercise	0.00	2.17	1.47	1.61	0.56
**Fold Change in BrdU relative between harvest times within the same genotype and condition ((DPI – previous DPI)/ previous DPI)**						
**Genotype**	**Condition**	**1 HPI**	**1 DPI**	**5 DPI**	**14 DPI**	**28 DPI**
wild-type	Sedentary	0.00	0.83	0.46	-0.36	-0.63
wild-type	Exercise	0.00	1.35	0.10	-0.27	-0.58
dexras1$^{-/-}$	Sedentary	0.00	1.26	-0.05	-0.10	-0.51
dexras1$^{-/-}$	Exercise	0.00	2.17	-0.22	0.06	-0.40

Table S3. Antibody concentrations for immunofluorescence, immunohistochemistry and western blotting.

Antibody	Dilution	Cat. No.	Company
rat anti-BrdU/ CldU	1:4,000 (BrdU IHC)<br>1:1,000 (BrdU/ CldU IF)	OBT0030G	Bio-Rad Laboratories
mouse monoclonal anti-BrdU	1:2,000	347580	BD Biosciences
mouse monoclonal anti-IdU	1:4,000	32D8.D9	Novus Bio
rat anti-Ki-67	1:1,000	SolA15	eBioscience
goat polyclonal anti-Doublecortin	1:100	C-18	Santa Cruz Biotechnology
mouse monoclonal anti-NeuN	1:1,000	A60	EMD Millipore
goat polyclonal anti-Sox2	1:1,000	Y-17x	Santa Cruz Biotechnology
mouse anti-GFAP	1:1,000	A21282	Life Technologies
rabbit anti-S100B	1:500	ab52642	Abcam
rabbit anti-p-CREB (Ser133)	1:100	9198S	Cell Signaling Technology
rabbit anti-NR2A	1:10,000	ab133265	Abcam
rabbit anti-NR2B	1:10,000	4207S	Cell Signaling Technology
rabbit anti-Phospho-p44/42 MAPK (T202/Y204)	1:5,000	9101S	Cell Signaling Technology
rabbit anti-p44/42 MAPK	1:1,000	9102S	Cell Signaling Technology
rabbit anti-actin	1:10,000		Sigma-Aldrich
anti-rat IgG or anti-rabbit IgG	1:300		Vector Laboratories
Alexa-Fluor secondary antibodies	1:1,000		Life Technologies
goat anti-Rabbit IgG H&L (HRP)	1:100,000		Life Technologies

Table S4. qPCR Primers

Gene	Forward	Reverse
bdnf	5’-AGCATGAAATCTCCCAGCCT-3’	5’-CGGTCCCCAAGGTTCTAGAC-3’
trkb	5’-CGCCCTGTGAGCTGAACTCTG-3’	5’-CTGCTTCTCAGCTGCCTGACC-3’
vegf-a	5’-CCAGGAGGACCTTGTGTGAT-3’	5’-GGGAAGGGAAGATGAGGAAG-3’
dexras1	5’-CCACACAACCTGAGGACCTT-3’	5’-TTCACAGCAGGTGACTGTCC-3’
gapdh	5’-TCCATGACAACTTTGGCATT-3’	5’-GTTGCTGTTGAAGTCGCAGG-3’
